# Magnetic resonance imaging-based radiomics was used to evaluate the level of prognosis-related immune cell infiltration in breast cancer tumor microenvironment

**DOI:** 10.1186/s12880-024-01212-9

**Published:** 2024-02-02

**Authors:** Hua Qian, Xiaojing Ren, Maosheng Xu, Zhen Fang, Ruixin Zhang, Yangyang Bu, Changyu Zhou

**Affiliations:** 1 Department of Radiology, The First Affiliated Hospital of Zhejiang Chinese Medical University (Zhejiang Provincial Hospital of Chinese Medicine), China , 54 Youdian Road, Hangzhou, 310006 Hangzhou China; 2 School of the First Clinical Medicine, Zhejiang Chinese Medical University, Hangzhou, China

**Keywords:** Breast cancer, Radiomics, Tumor microenvironment, M2 macrophages, Magnetic resonance imaging

## Abstract

**Purpose:**

The tumor immune microenvironment is a valuable source of information for predicting prognosis in breast cancer (BRCA) patients. To identify immune cells associated with BRCA patient prognosis from the Cancer Genetic Atlas (TCGA), we established an MRI-based radiomics model for evaluating the degree of immune cell infiltration in breast cancer patients.

**Methods:**

CIBERSORT was utilized to evaluate the degree of infiltration of 22 immune cell types in breast cancer patients from the TCGA database, and both univariate and multivariate Cox regressions were employed to determine the prognostic significance of immune cell infiltration levels in BRCA patients. We identified independent prognostic factors for BRCA patients. Additionally, we obtained imaging features from the Cancer Imaging Archive (TCIA) database for 73 patients who underwent preoperative MRI procedures, and used the Least Absolute Shrinkage and Selection Operator (LASSO) to select the best imaging features for constructing an MRI-based radiomics model for evaluating immune cell infiltration levels in breast cancer patients.

**Results:**

According to the results of Cox regression analysis, M2 macrophages were identified as an independent prognostic factor for BRCA patients (HR = 32.288, 95% CI: 3.100–357.478). A total of nine significant features were selected to calculate the radiomics-based score. We established an intratumoral model with AUCs (95% CI) of 0.662 (0.495–0.802) and 0.678 (0.438–0.901) in the training and testing cohorts, respectively. Additionally, a peritumoral model was created with AUCs (95% CI) of 0.826 (0.710–0.924) and 0.752 (0.525–0.957), and a combined model was established with AUCs (95% CI) of 0.843 (0.723–0.938) and 0.744 (0.491–0.965). The peritumoral model demonstrated the highest diagnostic efficacy, with an accuracy, sensitivity, and specificity of 0.773, 0.727, and 0.818, respectively, in its testing cohort.

**Conclusion:**

The MRI-based radiomics model has the potential to evaluate the degree of immune cell infiltration in breast cancer patients, offering a non-invasive imaging biomarker for assessing the tumor microenvironment in this disease.

**Supplementary Information:**

The online version contains supplementary material available at 10.1186/s12880-024-01212-9.

## Introduction

Breast cancer is a prevalent cancer worldwide and the second leading cause of cancer-related deaths [1]. In recent years, it has become increasingly evident that breast cancer involves not only tumor cells but significant changes in the surrounding tumor microenvironment (TME) as well. These changes are now recognized as crucial factors in the development and progression of breast cancer, as well as potential targets for treatment. The TME comprises proliferating tumor cells and a range of non-cancerous cells, including fibroblasts, immune cells, endothelial cells, infiltrating inflammatory cells, adipocytes, as well as signaling molecules and extracellular matrix (ECM) components [2]. Tumor cells interact symbiotically with multiple cellular components of the TME to form a more complex organoid structure than normal healthy tissue, with the tumor immune microenvironment consisting of immune-infiltrating cells becoming a significant area of research [3–5]. Studies have indicated that tumor infiltration lymphocytes (TILs), dendritic cells (DC), tumor-associated macrophages (TAM), tumor-associated neutrophils (TAN), and many other cells have relevance to tumor treatment and prognosis [6–8].

Currently, the assessment of immune infiltration typically requires tissue samples obtained post-surgery. However, the dynamic nature of the immune response means that non-invasive methods for evaluating the tumor microenvironment (TME) would be helpful, providing the ability to assess immune infiltration throughout the course of treatment [9]. Therefore, the establishment of a validated system for the in vivo evaluation of the immune microenvironment has become an urgent issue. “Radiogenomics” explores links between imaging phenotypes (image data) and disease genotypes (genomic patterns) [10, 11]. Two data resources, The Cancer Genome Atlas (TCGA) and the Cancer Imaging Archive (TCIA), provide cancer genome profiles and medical imaging information, respectively, to facilitate interdisciplinary research, including imaging genomic studies [12–14]. An important advantage of MRI over molecular data obtained by biopsy is that imaging provides a global, unbiased view of the entire tumor and its surrounding tissue. In addition to visual assessment by radiologists, quantitative image analysis may reveal other useful biomarkers in cancer [15–18]. Furthermore, tumor biology changes over time, and treatment may lead to alterations in the tumor immune microenvironment. As a result, imaging-based biomarkers can be beneficial for non-invasive and systemic quantification of the expression of immune-related parameters.

In this study, we utilized the CIBERSORT algorithm [19] to estimate the degree of infiltration of 22 immune cell types from RNA sequencing (RNA-seq) data in a cohort of BRCA patients. Our goal was to establish MRI-based radiomic features to non-invasively evaluate the level of immune cell infiltration.

## Materials and methods

### Genetic data acquisition and immune cell correlation analysis

The TCGA database (https://portal.gdc.cancer.gov/) [12], which is the largest database of cancer genetic information available, contains data on gene expression, miRNA expression, copy number variants, DNA methylation, SNPs, and more. For this study, we downloaded the raw mRNA expression data of processed BRCA, including a normal group (*n* = 113) and a tumor group (*n* = 1109). RNA-seq data from various subgroups of patients were analyzed using the CIBERSORT algorithm to infer the relative proportions of the 22 immune infiltrating cells. We performed Spearman correlation analysis on gene expression levels and immune cell content, with *p < 0.05* considered statistically significant. After excluding the samples without survival information, 1089 patients were obtained after shortening the long sample. Univariate and multivariate Cox regression analyses (survival time and survival status were used for two dependent variables) were applied to evaluate the prognostic value of immune cell infiltration levels in BRCA patients, and independent prognostic factors were identified via Cox regression analysis.

### MRI acquisition and Radiomics features extraction

**Fig. 1:**
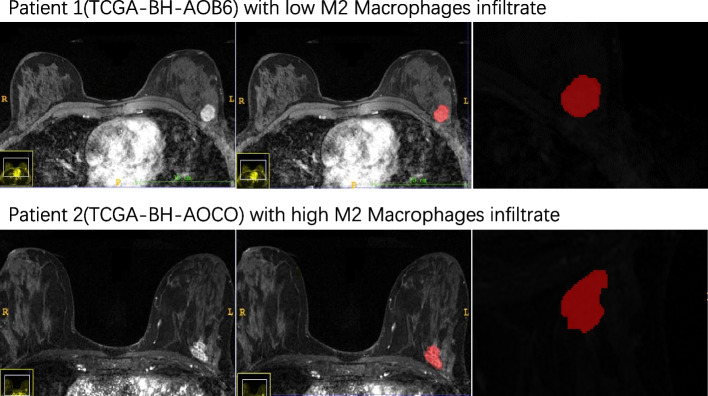
The volume of interest (VOI) from MRI data of two patients from two different groups

For this study, we analyzed breast cancer imaging data from the TCGA, using the following inclusion criteria: (1) complete clinical information on breast cancer，including age and sex, pathological classification, TNM stage, clinical stage, immunohistochemistry(IHC)type; (2) complete MRI data in axial position，including T2-weighted imaging, diffusion weighted imaging, T1 -weighted imaging and dynamic contrast-enhanced magnetic resonance imaging, excluding those with only sagittal enhanced images and images with low resolution. The second phase of the enhanced images (arterial phase) is selected to extract the radiomic features; and (3) oncogene expression data from RNA sequencing and mutation data from whole exome sequencing. A total of 73 patients were recruited, and both clinical and imaging data are publicly available from the Cancer Imaging Archive (TCIA) (www.cancerimagingarchive.net) [13]. Detailed imaging protocols for the TCGA cohort have been reported elsewhere [20]. In summary, scans were performed between September 1999 and June 2006 at three centers using 1.5-T or 3.0-T GE Healthcare, Siemens or Philips whole-body MRI systems with standard double breast coils. The volume of interest (VOI) from MRI data was manually segmented using ITK-SNAP (version 3.80; http://www.itksnap.org) [21], with a breast MRI radiologist (5 years of experience) completing the delineation of the lesions. The VOI was segmented layer by layer along the perimeter of the tumor contour on the DCE image (arterial phase at the second scan) (Fig. 1). An internal procedure implemented in Deepwise Multimodal Research Platform (version 1.6.3.6; http://keyan.deepwise.com/) was used to obtain infiltrative margin data, with a ring formed around the primary tumor and the tumor margin automatically expanded outwards by 5 mm [22] to obtain a volume of interest in the peritumor region (peritumor VOI) (large vascular systems, adjacent organs, and gases were excluded). Afterward, a second breast MRI radiologist (22 years of experience) reviewed all VOIs segmented by the first radiologist. Intraobserver and interobserver intraclass correlation coefficients (ICCs) were calculated by radiologists A and B, and features with ICC > 0.75 for both intraobserver and interobserver agreement were considered repeatable and used for further feature selection.

A total of 1648 radiomics features, including first-order features, shape features, and texture features such as grey-level co-occurrence matrix (GLCM), grey-level travel matrix (GLRLM), grey-level size zone matrix (GLSZM), and grey-level dependence matrix (GLDM), were extracted using the Pyradiomics package (version 3.0.1; https://www.radiomics.io/pyradiomics.html; Python [version 3.7.3; https://www.python.org/down loads/]) after logarithmic and wavelet filtering [23]. All radiomics features were normalized by zero-mean (i.e., subtracted from the mean and divided by the standard deviation) and the sample was randomly divided into training and testing cohorts in a ratio of 7:3. The important radiomics features of each model were then screened using the LASSO method.

### Predictive model construction based on Radiomics

To investigate the correlation between the level of immune cell infiltration and disease prognosis, we first classified the level of immune cell infiltration into high and low infiltration levels based on median values [24]. Next, we constructed a model using LASSO-logistics regression analysis, based on selected peritumoral radiomics features as the independent variable, and the level of immune cell infiltration as the dependent variable. We inferred the level of immune cell infiltration from the imaging histological features. The performance of each model was evaluated using the area under the curve (AUC) of the receiver operating characteristic curve (ROC). In addition to AUC, we calculated accuracy, sensitivity, and specificity and used calibration curves to evaluate the performance of each model.

### Statistical analysis

For statistical analysis, we used the SciPy package to test all features for Shapiro-Wilk normality. Variables meeting normality were subjected to analysis of variance and independent samples t-tests, while skewed features were subjected to Mann-Whitney U tests. LASSO (Least Absolute Shrinkage and Selection Operator) was performed using R software (version 4.1.1, https://www.r-project.org/). The calibration curve was used to evaluate the robustness of the models. We considered a two-sided *P* value *< 0.05* statistically significant.

## Results

### BRCA-associated immune infiltration mapping

**Fig. 2:**
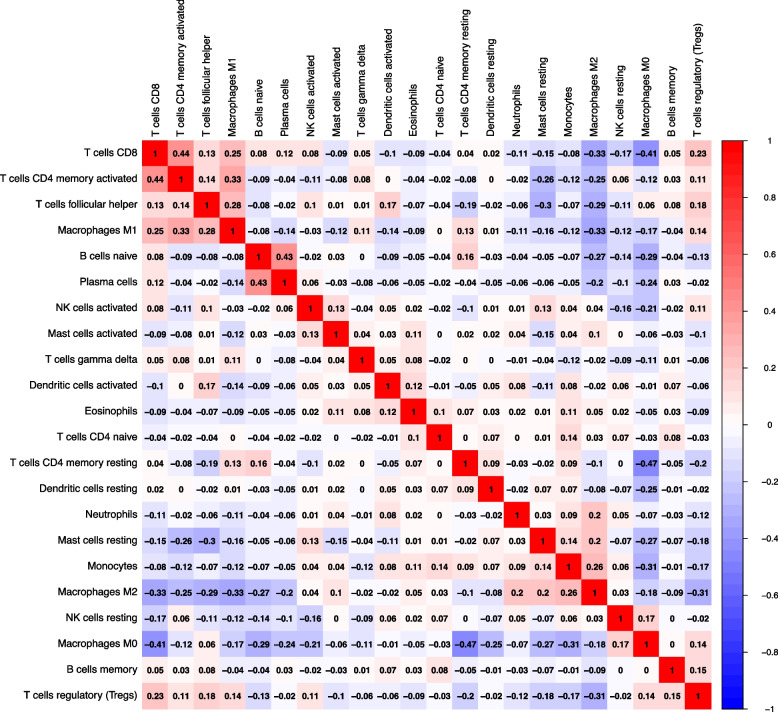
Correlation heat map showing the correlation of 22 types of immune cells estimated by CIBERSORT

The microenvironment is mainly composed of fibroblasts, immune cells, extracellular matrix, multiple growth factors, inflammatory factors and specific physicochemical features. The microenvironment significantly influences the diagnosis, survival outcome and clinical treatment sensitivity of the disease. By analyzing the relationship between key genes and immune infiltration, we further explore the potential mechanisms and key genes that have an impact on tumor progression. The immune cell correlation heat map is shown in Fig. 2. Red indicates positive correlation, blue indicates negative correlation. The darker the colour, the stronger the correlation. (using the R package “corrplot”).

**Fig. 3:**
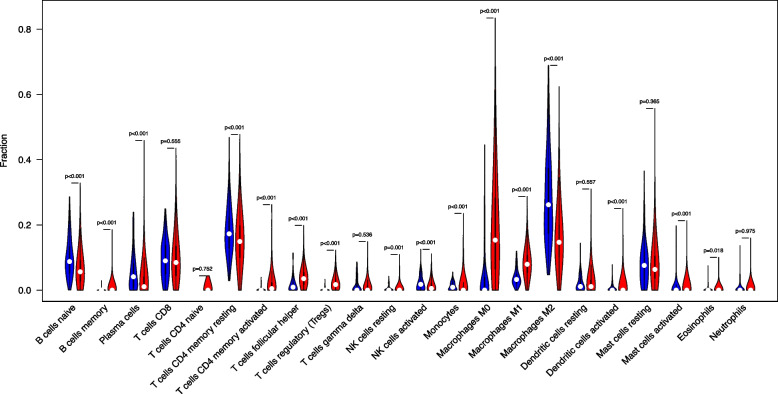
Violin plot showing the infiltration levels of 22 types of immune cells estimated by CIBERSORT

A comparative analysis of the microenvironment scores between the tumor and normal groups revealed that various immune microenvironment factors, including B cells naive, T cells CD4 memory resting, T cells follicular helper, T cells regulatory (Tregs), and Macrophages M0, were significantly different between the two groups. The normal group is represented in blue and the tumor group in red (Fig. 3), with the analysis being conducted using the R package “vioplot”.

### The level of immune cell infiltration is associated with the survival of breast cancer patients

**Fig. 4:**
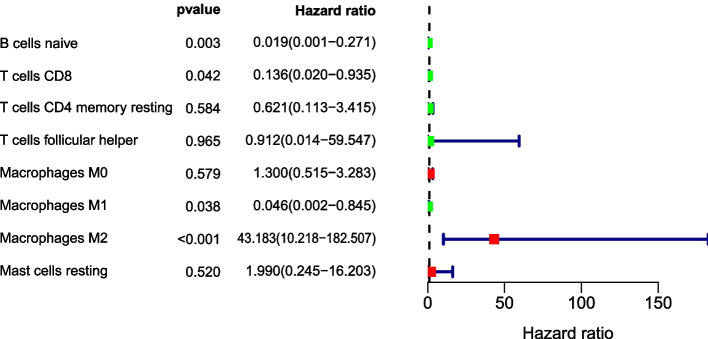
Univariate cox regression analysis of the forest plot, green represents the biological effect of inhibiting breast cancer, red represents the biological effect of promoting breast cancer, *p* < 0.05 is statistically significant, HR > 1 represents the inhibitory effect, HR < 1 represents the promoting effect of survival

**Fig. 5:**
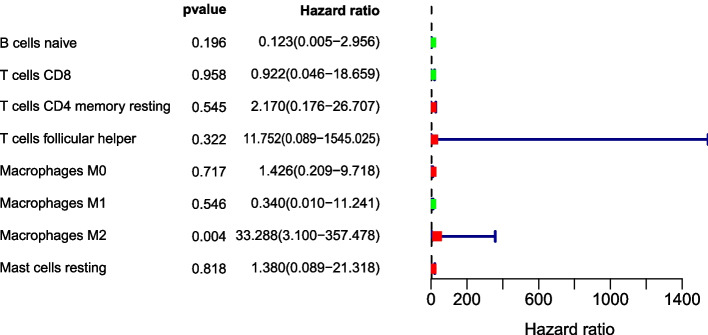
Multivariate cox regression analysis of forest plots, green represents the biological effect of inhibiting breast cancer, red represents the biological effect of promoting breast cancer, *p* < 0.05 is statistically significant, HR > 1 represents the inhibitory effect of survival, HR < 1 represents the promoting effect of survival

Clinical survival data for 1089 patients are presented in Supplementary Table 1. We utilized univariate and multivariate Cox regression analyses to evaluate the prognostic value of the level of immune cell infiltration in BRCA patients. A smaller *p*-value indicated a stronger association between immune cells and prognosis. An HR > 1 indicated high-risk immune cells, with higher expression levels correlating with worse prognoses. Univariate cox regression showed that the infiltration levels of B cell naive, T cells CD8, Macrophages M1, Macrophages M2 were associated with prognosis (*p* < 0.05), including B cell naive, T cells CD8, macrophages M1, macrophages M2. The higher the expression of Macrophages M1, the better the prognosis, while the higher the expression of Macrophages M2, the worse the prognosis (HR = 43.183). Multivariate cox regression analysis showed that only the infiltration level of Macrophages M2 cells was related to the prognosis (HR = 33.288, *p* < 0.05). Therefore, Macrophages M2 cells are an independent prognostic factor in BRCA patients, with higher expression indicating worse prognosis (Figs. 4 and 5), with the analysis being conducted using the R package “survival”.

### General information comparison

**Fig. 6:**
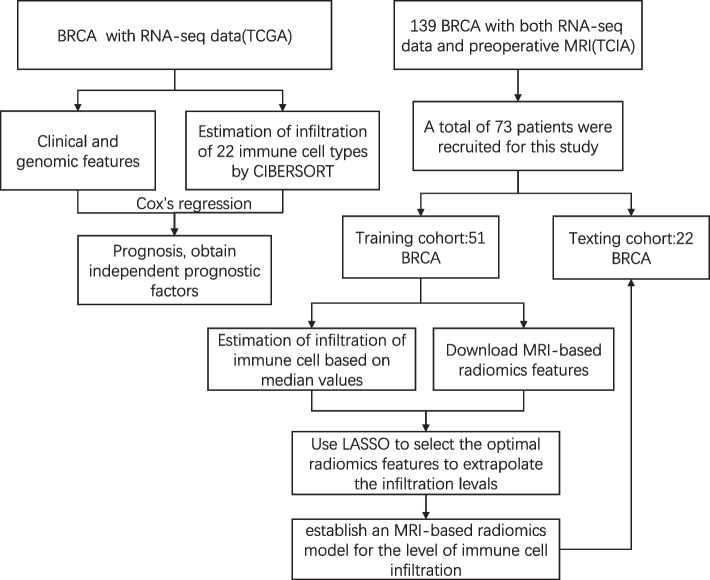
Patient survival analysis and the workflow for using radiomics to extrapolate the infiltration levels of immune cells

Figure 6 illustrates the patient survival analysis and the workflow for using radiomics to extrapolate the infiltration levels of immune cells. A total of 73 patients with preoperative MRI-based radiomic data from TCIA were selected from the BRCA patients with RNA-seq data for inclusion in this study. The median of M2 Macrophages infiltration levels for the 73 patients was 0.122, with 36 patients having high M2 Macrophages infiltration levels and 37 patients having low infiltration levels. The patients were randomly assigned to the training cohort (*n* = 51) and the testing cohort (*n* = 22). Table 1 presents the clinical and pathological characteristics of the 73 patients.

**Table 1:** Clinical and pathological Characteristics for 73 Patients

	Tumor with high M2 Macrophages infiltrate (*n* = 36,49.3%)	Tumor with low M2 Macrophages infiltrate (*n* = 37,50.7%)	*p value*^*a*^
Age			
Median	59	53	
Mean ± SD	58.0 ± 12.0	52.7 ± 11.2	
T			0.710
T1	15(45.5)	18(54.5)	
T2	19(54.3)	16(45.7)	
T3	2(40.0)	3(60.0)	
N			0.466
N0	17 (50.0)	17 (50.0)	
N1	14(48.3)	15(51.7)	
N2	1(20.0)	4(80.0)	
N3	3(75.0)	1(25.0)	
Nx^b^	1(100.0)	0(0)	
M			0.457
M0	23(46.0)	27(54.0)	
Mx^c^	13(56.5)	10(43.5)	
Stage			
I	9(47.4)	10(52.6)	0.843
II	23(52.3)	21(47.7)	
III	4(40.0)	6(60.0)	
Histological Type			1.000
Invasive Ductal Carcinoma	31(49.2)	32(50.8)	
Invasive Lobular Carcinoma	4(50.0)	4(50.0)	
Other	1(50.0)	1(50.0)	
ER			0.189
Positive	33(54.1)	29(45.9)	
Negative	3(27.3)	8(72.7)	
PR			0.794
Positive	27(50.9)	26(49.1)	
Negative	9(45.0)	11(55.0)	
HER2			0.646
Positive	7(58.3)	5(41.7)	
Negative	29(48.3)	31(51.7)	
Equivocal	0(0)	1(100.0)	
IHC type			0.288
HR+/HER2-	26(52.0)	24(48.0)	
HER2+	7(58.3)	5(41.7)	
ER−/PR−/HER2-	3(27.3)	8(72.7)	

*Abbreviations*: *T* the primary tumor, *N* regional lymph nodes, and *M* distant metastases, *ER* Estrogen receptor, *HER2* Human epidermal growth factor receptor 2, *HR* Hormone receptor, *PR* Progesterone receptor, *a* Fisher’s exact test, *b* Lymph node stage is not available, *c* Metastasis cannot be measured

## Construction of the radiomics model

Using LASSO, we selected eight peritumor high weight features and one intratumor high weight feature from a pool of 1648 features. These selected features included five first-order features, one grey-level correlation matrix feature, one grey-level region size matrix feature, and two grey-level travel matrix features. Table 2 provides a detailed list of the selected features, along with their corresponding coefficients.

**Table 2:** Names and Coefficients of High Weight Features

Name	Coefficients
(1) Names and Coefficients of High Weight Features Extracted from Peritumor	
square_firstorder_Minimum	− 0.481
wavelet.LLH_firstorder_Skewness	− 0.350
lbp.2D_glszm_ZoneVariance	−0.186
wavelet.LLL_firstorder_10Percentile	−0.098
squareroot_firstorder_10Percentile	−0.064
wavelet.HLH_gldm_DependenceVariance	0.071
wavelet.LLH_firstorder_Kurtosis	0.093
wavelet.LLH_glrlm_LongRunEmphasis	0.597
(2) Names and Coefficients of High Weight Features Extracted from Intratumor	
wavelet.HLL_glrlm_RunVarianc	0.389
(3) Names and Coefficients of High Weight Features Extracted from Combined	
Peri_square_firstorder_Minimum	−1.071
Peri_lbp.2D_glszm_ZoneVariance	−0.464
Peri_wavelet.LLH_firstorder_Skewness	−0.409
Peri_squareroot_firstorder_10Percentile	−0.168
Peri_wavelet.LLL_firstorder_10Percentile	−0.155
Peri_wavelet.HLH_gldm_DependenceVariance	0.132
Intra_wavelet.HLL_glrlm_RunVarianc	0.231
Peri_wavelet.LLH_firstorder_Kurtosis	0.257
Peri_wavelet.LLH_glrlm_LongRunEmphasis	0.784

**Fig. 7:**
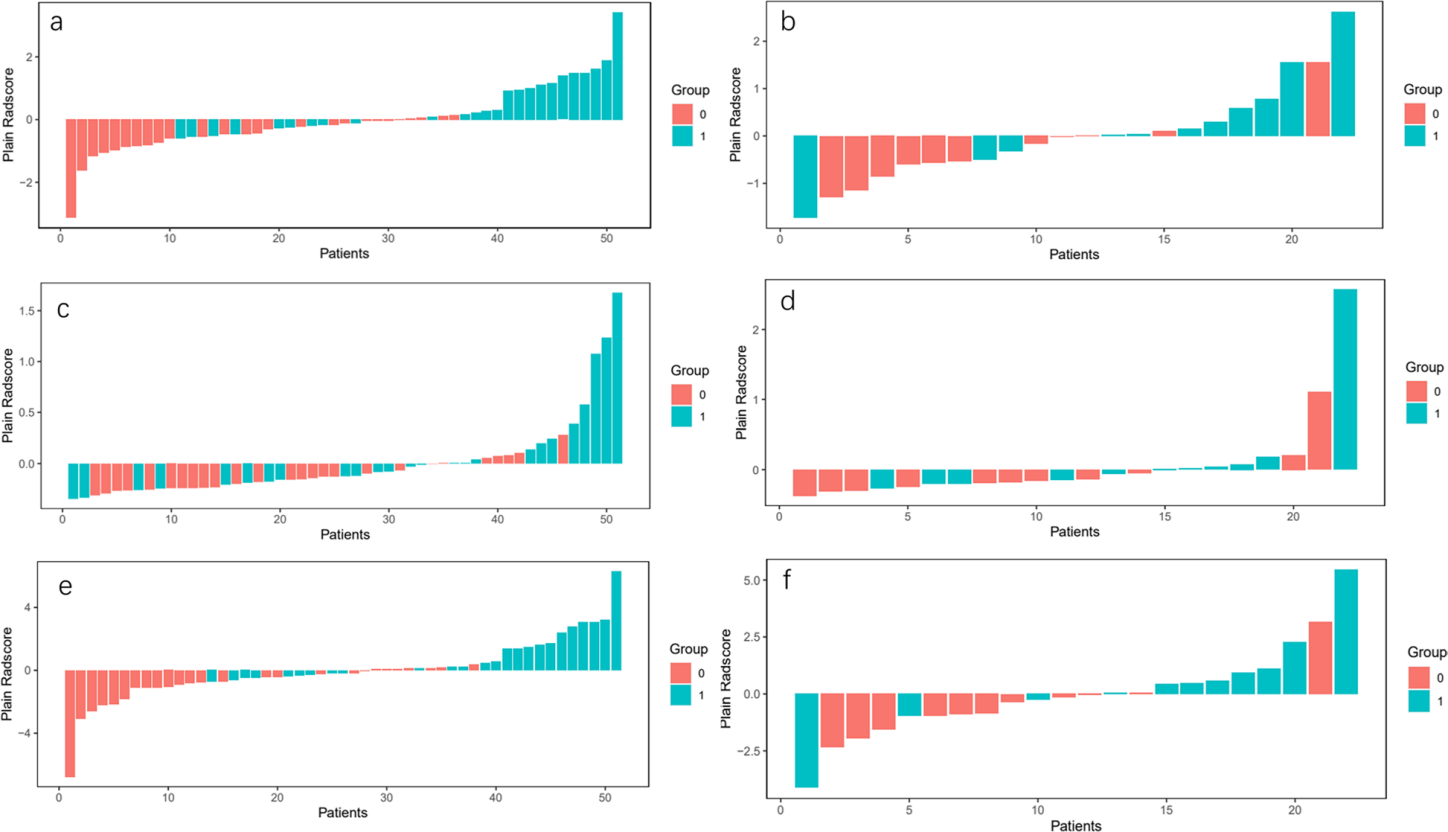
The waterfall plots of radscore for training(**A**), testing(**B**) in peritumoral model; training(**C**), testing(**D**) in intratumoral model; training(**E**), testing(**F**) in combined model

**Fig. 8:**
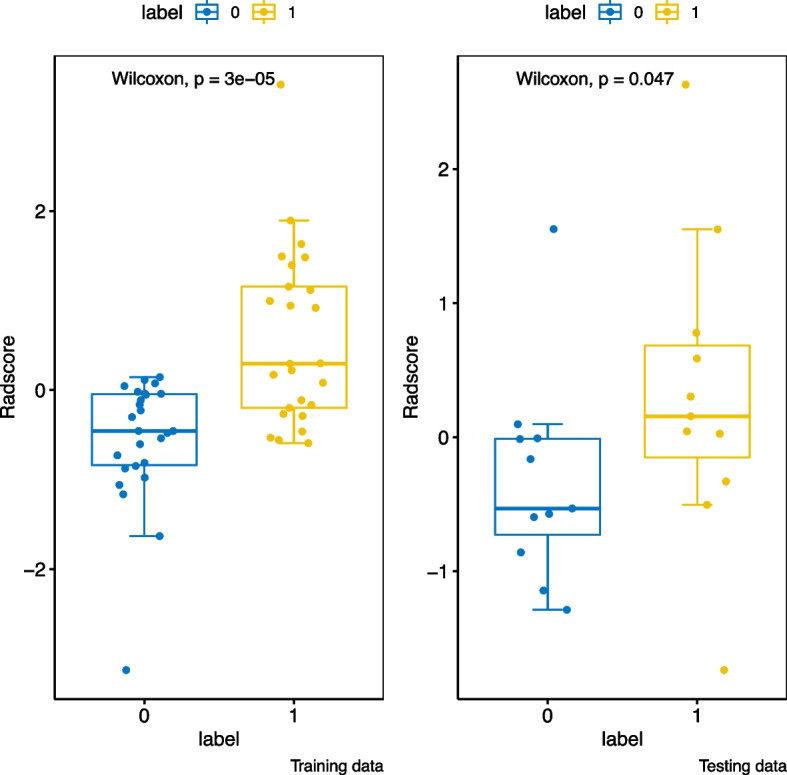
The box plot of radscore for training and testing cohort in peritumoral model

We calculated Radscore scores for both the training and testing cohorts. We then plotted waterfall plots of Radscore (Fig. 7) for each cohort to differentiate between low infiltration levels (Group 0) and high infiltration levels (Group 1) of Macrophages M2 cells in the immune microenvironment of breast cancer. Additionally, a box plot of Radscore in the peritumoral model is shown in Fig. 8. The Radscore was calculated as the product of the characteristic coefficient and its corresponding value.

**Fig. 9:**
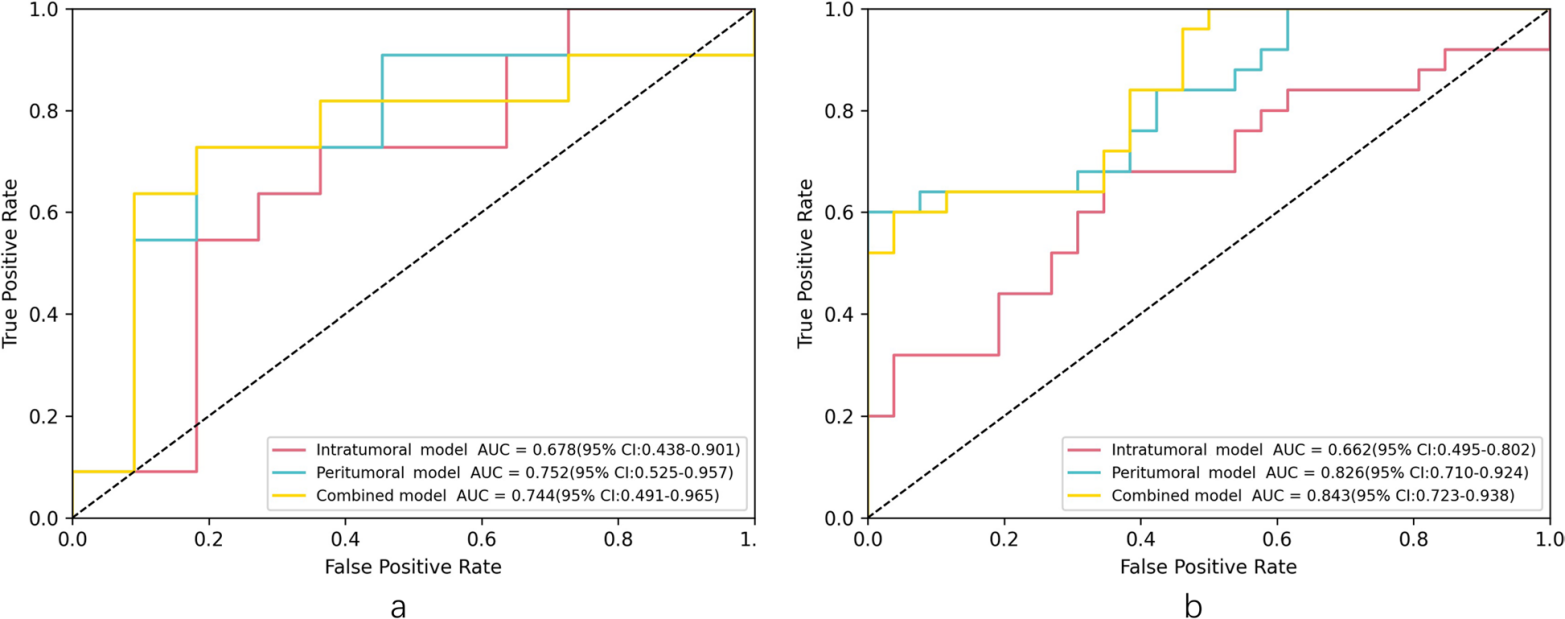
The ROC for training(**a**), testing(**b**) in three models

**Fig. 10:**
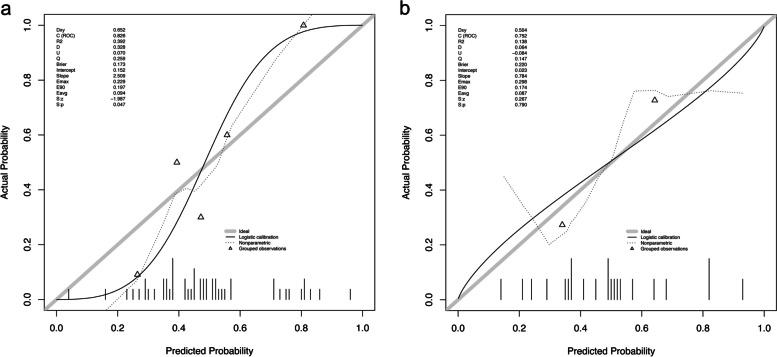
The calibration curve for training (**a**), testing(**b**) in peritumoral model

We used nine imaging features to establish predictive models for the level of M2 Macrophages infiltration in the immune microenvironment of breast cancer, based on the LASSO-logistics regression analysis method. We plotted ROC curves (Fig. 9) to evaluate the diagnostic performance of the predictive individual models. We calculated AUCs, accuracy, sensitivity, and specificity for each model (Table 3) and found that the peritumor model had better predictive performance than the other two models. The calibration curves for each model showed good agreement between the predicted and actual values, with no statistical difference between the Hosmer-Lemeshow test (*p* > 0.05), indicating that the predicted and actual results were in good agreement. Figure 10 shows the calibration curves for one of the peritumoral models.

**Table 3:** Model evaluation of three classification models for predicting Macrophages M2 cell infiltration

Cohort	Model	AUC (95% CI)	Accuracy	Sensitivity	Specificity
Training	intratumoral	0.662 (0.495–0.802)	0.667	0.680	0.654
	peritumoral	0.826 (0.710–0.924)	0.804	0.600	1.000
	combined	0.843 (0.723–0.938)	0.784	0.600	0.962
Testing	intratumoral	0.678 (0.438–0.901)	0.682	0.636	0.727
	peritumoral	0.752 (0.525–0.957)	0.773	0.727	0.818
	combined	0.744 (0.491–0.965)	0.773	0.636	0.909

## Discussion

In this study, we established MRI-based radiomics features to evaluate the level of tumor immune cell infiltration in breast cancer patients. We also identified specific immune cell types whose infiltration levels correlated with the prognosis of these patients. Our findings suggest that radiomics could serve as a valid and non-invasive method for assessing the immune status of breast cancer patients. Overall, our study highlights the potential of radiomics in improving the diagnosis and prognosis of breast cancer, and may pave the way for more personalized and effective cancer management strategies.

Recent studies have highlighted the crucial role of the tumor microenvironment (TME) in cancer patients. The immune microenvironment of the tumor is a key determinant of treatment efficacy. In this study, we utilized CIBERSORT, an algorithm that analyses RNA sequencing data to estimate the proportion of immune cells, to calculate the level of infiltration of 22 immune cells in breast cancer. The algorithm provides an alternative to immunostaining and flow or mass cytometry-based methods, streamlining the analysis process [19]. Our study revealed that breast cancer patients have altered levels of multiple immune cell infiltration compared to healthy individuals. Several immune microenvironment factors, such as B cells naive, T cells CD4 memory resting, T cells follicular helper, T cells regulatory (Tregs), and Macrophages M0, were significantly different in breast cancer. Importantly, we found an independent negative prognostic value for M2 macrophage infiltration levels, consistent with previous studies [25]. Macrophages are the main immune cell type that infiltrates the tumor microenvironment. In the microenvironment, they are polarized into M1 or M2 subtypes and perform a variety of functions such as tissue development and homeostasis, inflammation, pathogen clearance and wound healing [26]. M1/M2 dysregulation is critical in tumor development, immune escape, and subsequent metastasis. M2 macrophages promote tumor growth, while M1 macrophages inhibit proliferation [27], and macrophage infiltration and PD-L1 expression on infiltrating macrophages can inhibit tumor responsive T cells, leading to resistance to immune checkpoint blockade therapy [28]. Therefore, the development and treatment of antitumor drugs that target macrophage polarization are a current therapeutic priority. Overall, our findings emphasize the importance of understanding the immune microenvironment in breast cancer and suggest potential avenues for future treatment strategies.

Although MRI is a routine screening method for breast cancer patients, until now, it has only provided detailed images of organs and tissues and not TME. Our study, however, found that MRI-based radiomics features can reveal the level of infiltration of M2 macrophages in the TME of breast cancer patients. Previous literature has also indicated that radiomics can predict the level of immune cell infiltration in tumor patients. Sun et al. constructed a CT-based radiolabeling, which included eight variables to assess CD8+ T cell infiltration determined by RNA-seq data [29]. Additionally, other scholars have evaluated the degree of glioma immune cell infiltration by constructing a preoperative T2-weighted MRI-based radiomics model [30]. Radiomics has the advantage of non-invasive monitoring of TME and may become a new biomarker for predicting response to immunotherapy.

In our study, we extracted one intratumor feature and eight peritumor features, suggesting that peritumor features better reflect the level of M2 macrophage infiltration. The peritumoral radscore box plot indicates that larger radscore values correspond to higher levels of infiltration, and higher levels of M2 macrophage infiltration are biomarkers of poor prognosis in breast cancer. Previous studies have found that the peritumor secretes a large number of growth factors and cytokines that induce hypoxia and angiogenesis, and play an important role in tumor development, progression, or metastasis. Therefore, the integration of combined model features can provide a more comprehensive reflection of the aggressive and metastatic features of the tumor [31, 32]. Our study confirmed that both the peritumor model and combined model are better at discriminating between low and high levels of M2 macrophage infiltration. However, peritumoral imaging features that reflect the level of M2 macrophage infiltration are more robust, providing additional clues for our future studies.

Our study has several limitations that need to be addressed. Firstly, the size of our patient cohort is relatively small, and the number of available breast cancer MRI images in the TCIA database and RNA information available in the TCGA is limited. Secondly, the scanning machines and protocols used at the time were not as advanced as they are now, and the use of data from three different centers requires more sophisticated methods to reduce inconsistencies in radiomics features. Additionally, the level of immune cell infiltration was only evaluated in terms of high and low and was not quantified, and the relationship between quantified values and prognosis remains to be further investigated. Furthermore, the accuracy of imaging features in predicting the level of immune cell infiltration requires further external validation. We have only extracted enhanced second-stage MRI images and have not developed a model incorporating clinical features. Future studies with multiple sequences of imaging features are necessary to select the best model. Lastly, this is a retrospective analysis, and a multicenter prospective study with a larger dataset is needed to confirm our findings.

In summary, our study demonstrates that a breast MRI-based radiomics model has the potential to non-invasively assess the level of TME, particularly M2 macrophage infiltration. We also found that peri-tumoral radiomics features better reflect the level of immune cell infiltration. While this study requires a larger cohort for further evaluation, it provides potential value for radiomics as a non-invasive imaging biomarker in the clinical prognosis of breast cancer patients. Further studies are needed to quantify the level of immune cell infiltration using radiomics features from larger samples.

### Supplementary Information

**Additional file 1.**

## Data Availability

Clinical and imaging data are publicly available from TCIA (www.cancerimagingarchive.net/). For the TCGA breast cancer cohort, gene expression data derived from RNA-seq and mutational data derived from. whole-exome sequencing are available in the Genomic Data Commons (https://portal.gdc.cancer.gov/).
