# Supplementary material for: Magnetic resonance imaging-based radiomics was used to evaluate the level of prognosis-related immune cell infiltration in breast cancer tumor microenvironment
Source: BMC Med Imaging. 2024 Feb 2;24:31. doi: 10.1186/s12880-024-01212-9 (PMC10835863; doi:10.1186/s12880-024-01212-9)
Supplement: Supplementary file 1 — Additional file 1. [file 12880_2024_1212_MOESM1_ESM.docx]

**Supplement Table：Follow-up time, survival status and macrophage infiltration values of 1089 patients**

| Id | Futime（d) | Fustat | Macrophages M2 |
| --- | --- | --- | --- |
| TCGA-E2-A1IH | 1026 | 0 | 0.0833634 |
| TCGA-E2-A15I | 1692 | 0 | 0.088147735 |
| TCGA-BH-A0E2 | 435 | 0 | 0.066058739 |
| TCGA-AC-A2QI | 588 | 0 | 0.172320632 |
| TCGA-B6-A408 | 2072 | 0 | 0.136194313 |
| TCGA-A2-A4RX | 742 | 0 | 0.069231919 |
| TCGA-BH-A0W7 | 1363 | 0 | 0.095965326 |
| TCGA-AR-A24O | 2561 | 0 | 0.135698168 |
| TCGA-A8-A092 | 942 | 0 | 0.250403407 |
| TCGA-B6-A0X7 | 1781 | 1 | 0.137366808 |
| TCGA-BH-A18L | 811 | 1 | 0.303156938 |
| TCGA-A2-A04R | 3709 | 0 | 0.034304959 |
| TCGA-E9-A1NA | 1112 | 0 | 0.214268736 |
| TCGA-BH-A209 | 3959 | 1 | 0.093531677 |
| TCGA-D8-A13Y | 1728 | 0 | 0.144490455 |
| TCGA-B6-A0RP | 3126 | 1 | 0.193340306 |
| TCGA-BH-A1FR | 1642 | 1 | 0.194197994 |
| TCGA-AO-A03V | 1351 | 0 | 0.061460409 |
| TCGA-C8-A27B | 439 | 0 | 0.089763312 |
| TCGA-A2-A1FZ | 683 | 0 | 0.175116017 |
| TCGA-D8-A1JG | 1612 | 0 | 0.19012127 |
| TCGA-A2-A0EV | 968 | 0 | 0.16182354 |
| TCGA-B6-A0RE | 7777 | 0 | 0.104143574 |
| TCGA-AC-A2FF | 2420 | 0 | 0.142556908 |
| TCGA-BH-A0C0 | 1270 | 0 | 0.163163082 |
| TCGA-D8-A1JM | 590 | 0 | 0.052431729 |
| TCGA-S3-AA10 | 468 | 0 | 0.061509101 |
| TCGA-A2-A25D | 0 | 0 | 0.108679337 |
| TCGA-A2-A0SX | 1534 | 0 | 0.06036378 |
| TCGA-BH-A0BD | 554 | 0 | 0.113817553 |
| TCGA-A2-A0CY | 1673 | 0 | 0.146082845 |
| TCGA-EW-A6S9 | 463 | 0 | 0.090358378 |
| TCGA-A2-A4S2 | 643 | 0 | 0.165155983 |
| TCGA-D8-A27T | 398 | 0 | 0.11589272 |
| TCGA-AR-A24Z | 2403 | 0 | 0.324122905 |
| TCGA-AR-A0TW | 3009 | 0 | 0.144226431 |
| TCGA-A2-A0EN | 4088 | 0 | 0.136068774 |
| TCGA-AO-A12D | 2515 | 0 | 0.162159329 |
| TCGA-BH-A28Q | 1119 | 0 | 0.156213653 |
| TCGA-D8-A1XL | 606 | 0 | 0.156152912 |
| TCGA-AR-A24P | 84 | 0 | 0.167981931 |
| TCGA-C8-A130 | 370 | 0 | 0.177676407 |
| TCGA-E9-A1RF | 200 | 0 | 0.241898766 |
| TCGA-E2-A15S | 428 | 0 | 0.146966754 |
| TCGA-AR-A1AY | 1026 | 0 | 0.098638314 |
| TCGA-BH-A0HN | 516 | 0 | 0.40927232 |
| TCGA-PE-A5DD | 1953 | 0 | 0.065470104 |
| TCGA-AO-A03N | 2031 | 0 | 0.09385864 |
| TCGA-A7-A26H | 724 | 0 | 0.134460375 |
| TCGA-D8-A1Y2 | 433 | 0 | 0.123830196 |
| TCGA-GM-A2DD | 2282 | 0 | 0.132599368 |
| TCGA-BH-A0H0 | 461 | 0 | 0.188283002 |
| TCGA-E2-A15J | 1640 | 0 | 0.299121681 |
| TCGA-AC-A8OQ | 34 | 0 | 0.09776872 |
| TCGA-BH-A0H9 | 1247 | 0 | 0.197741356 |
| TCGA-AQ-A0Y5 | 172 | 1 | 0.338884361 |
| TCGA-A8-A06Y | 791 | 0 | 0.186798998 |
| TCGA-B6-A0RO | 4929 | 0 | 0.314131959 |
| TCGA-A8-A08A | 30 | 0 | 0.212644888 |
| TCGA-AC-A2QJ | 446 | 1 | 0.408723271 |
| TCGA-AR-A2LN | 997 | 0 | 0.170341671 |
| TCGA-EW-A3U0 | 532 | 0 | 0.076612507 |
| TCGA-A7-A0D9 | 1139 | 0 | 0.235193392 |
| TCGA-GM-A5PX | 551 | 0 | 0.12614151 |
| TCGA-AO-A0J7 | 618 | 0 | 0.010879433 |
| TCGA-AN-A0AM | 5 | 0 | 0.18776828 |
| TCGA-AN-A0FK | 213 | 0 | 0.13415921 |
| TCGA-A8-A0A2 | 579 | 0 | 0.184820165 |
| TCGA-BH-A1FC | 3472 | 1 | 0.073433375 |
| TCGA-EW-A1J5 | 477 | 0 | 0.151433075 |
| TCGA-BH-A0DL | 2381 | 0 | 0.161492845 |
| TCGA-EW-A1P0 | 1251 | 0 | 0.132262358 |
| TCGA-AN-A041 | 7 | 0 | 0.178719631 |
| TCGA-C8-A12Y | 1476 | 0 | 0.150862557 |
| TCGA-AR-A0TZ | 3262 | 1 | 0.251782541 |
| TCGA-E2-A159 | 762 | 0 | 0.154684637 |
| TCGA-BH-A0DT | 2403 | 0 | 0.047332001 |
| TCGA-E9-A247 | 828 | 0 | 0.170477945 |
| TCGA-AR-A24W | 1550 | 0 | 0.140592799 |
| TCGA-E2-A150 | 1935 | 0 | 0.10218525 |
| TCGA-AO-A1KT | 541 | 0 | 0.303808841 |
| TCGA-E2-A1B6 | 867 | 0 | 0.034217084 |
| TCGA-AR-A24M | 2947 | 0 | 0.213724232 |
| TCGA-A2-A1G0 | 616 | 0 | 0.087404003 |
| TCGA-A2-A0T0 | 533 | 0 | 0.129286866 |
| TCGA-AO-A12B | 2989 | 0 | 0.286253077 |
| TCGA-AQ-A1H2 | 475 | 0 | 0.439764167 |
| TCGA-AR-A0TP | 3386 | 0 | 0.185919018 |
| TCGA-JL-A3YX | 352 | 0 | 0.135027585 |
| TCGA-AC-A8OR | 40 | 0 | 0.330159073 |
| TCGA-OL-A5D7 | 1780 | 0 | 0.07896128 |
| TCGA-A2-A0ES | 2190 | 0 | 0.178299306 |
| TCGA-BH-A18U | 1563 | 1 | 0.223099068 |
| TCGA-AO-A0JC | 1547 | 0 | 0.065343303 |
| TCGA-D8-A1XT | 506 | 0 | 0.242677178 |
| TCGA-A8-A095 | 1277 | 0 | 0.164376118 |
| TCGA-C8-A1HL | 317 | 0 | 0.27363392 |
| TCGA-D8-A27L | 499 | 0 | 0.146903458 |
| TCGA-AR-A2LJ | 1865 | 0 | 0.147018093 |
| TCGA-S3-A6ZG | 399 | 0 | 0.158034098 |
| TCGA-GM-A2DB | 2406 | 0 | 0.059283252 |
| TCGA-AR-A5QN | 488 | 0 | 0.206284453 |
| TCGA-A2-A0YD | 769 | 0 | 0.147118457 |
| TCGA-HN-A2OB | 1883 | 0 | 0.196947022 |
| TCGA-E2-A1LA | 748 | 0 | 0.108217362 |
| TCGA-B6-A0WY | 3461 | 1 | 0.128623929 |
| TCGA-C8-A3M7 | 1034 | 1 | 0.019959241 |
| TCGA-A7-A6VY | 266 | 0 | 0.136668666 |
| TCGA-OL-A5S0 | 620 | 0 | 0.152748001 |
| TCGA-B6-A3ZX | 1152 | 1 | 0.033952691 |
| TCGA-EW-A1J2 | 403 | 0 | 0.165575104 |
| TCGA-BH-A18T | 224 | 1 | 0.135588399 |
| TCGA-C8-A8HP | 396 | 0 | 0.152021204 |
| TCGA-A2-A1FW | 528 | 0 | 0.225698955 |
| TCGA-BH-A0HI | 620 | 0 | 0.166181033 |
| TCGA-D8-A1JP | 639 | 0 | 0.29198803 |
| TCGA-GM-A3XL | 2108 | 0 | 0.167047586 |
| TCGA-D8-A1XU | 395 | 0 | 0.210740062 |
| TCGA-A2-A04Q | 2385 | 0 | 0.091211109 |
| TCGA-A2-A25B | 1291 | 0 | 0.226912686 |
| TCGA-UL-AAZ6 | 518 | 0 | 0.316857364 |
| TCGA-LL-A7T0 | 376 | 0 | 0.135983646 |
| TCGA-B6-A0RG | 2082 | 0 | 0.109724082 |
| TCGA-B6-A401 | 2596 | 0 | 0.065017945 |
| TCGA-W8-A86G | 347 | 0 | 0.198055891 |
| TCGA-D8-A1JE | 575 | 0 | 0.441554383 |
| TCGA-AR-A1AH | 3807 | 0 | 0.141376044 |
| TCGA-AC-A5EH | 511 | 0 | 0.173779107 |
| TCGA-B6-A0RM | 2373 | 1 | 0.086381059 |
| TCGA-A8-A075 | 518 | 0 | 0.230480919 |
| TCGA-B6-A0IJ | 7106 | 0 | 0.101323389 |
| TCGA-BH-A18I | 1093 | 0 | 0.06752503 |
| TCGA-AC-A3TN | 456 | 0 | 0.167645944 |
| TCGA-AO-A03T | 2124 | 0 | 0.090017461 |
| TCGA-A7-A426 | 364 | 0 | 0.102538665 |
| TCGA-AC-A2FO | 2255 | 0 | 0.115341332 |
| TCGA-S3-A6ZF | 502 | 0 | 0.126429504 |
| TCGA-LL-A73Y | 477 | 0 | 0.052182504 |
| TCGA-A2-A1G4 | 595 | 0 | 0.179601142 |
| TCGA-C8-A1HN | 394 | 0 | 0.287723991 |
| TCGA-A2-A0T4 | 624 | 0 | 0.107575513 |
| TCGA-D8-A27N | 519 | 0 | 0.196199031 |
| TCGA-LQ-A4E4 | 849 | 0 | 0.325714414 |
| TCGA-A2-A0ET | 1066 | 0 | 0.152700812 |
| TCGA-E9-A1R6 | 339 | 0 | 0.219270112 |
| TCGA-WT-AB44 | 883 | 0 | 0.171977723 |
| TCGA-A8-A07B | 1308 | 0 | 0.302278701 |
| TCGA-E2-A154 | 591 | 0 | 0.342360259 |
| TCGA-E2-A15L | 626 | 0 | 0.267086274 |
| TCGA-D8-A1XS | 496 | 0 | 0.265496663 |
| TCGA-AC-A3W6 | 0 | 0 | 0.120382018 |
| TCGA-D8-A1XJ | 664 | 0 | 0.288859966 |
| TCGA-AO-A0JA | 655 | 0 | 0.263636569 |
| TCGA-E2-A153 | 707 | 0 | 0.26935156 |
| TCGA-E9-A3Q9 | 560 | 0 | 0.241404337 |
| TCGA-BH-A0DS | 78 | 0 | 0.109246228 |
| TCGA-E2-A1B1 | 2653 | 0 | 0.122099963 |
| TCGA-D8-A3Z5 | 1015 | 0 | 0.123747163 |
| TCGA-AO-A1KS | 350 | 0 | 0.232432803 |
| TCGA-A2-A1FX | 1847 | 0 | 0.186699282 |
| TCGA-A8-A0AB | 518 | 0 | 0.22432504 |
| TCGA-E9-A1RE | 1054 | 0 | 0.097787232 |
| TCGA-A2-A0T3 | 1516 | 0 | 0.130522001 |
| TCGA-EW-A1OY | 908 | 0 | 0.137413064 |
| TCGA-GM-A3XN | 2019 | 0 | 0.055843162 |
| TCGA-E2-A109 | 1417 | 0 | 0.084006314 |
| TCGA-BH-A0AZ | 1919 | 0 | 0.121946159 |
| TCGA-JL-A3YW | 360 | 0 | 0.146126402 |
| TCGA-LL-A7SZ | 594 | 0 | 0.16371758 |
| TCGA-BH-A0EB | 745 | 0 | 0.175376354 |
| TCGA-E2-A2P6 | 1051 | 0 | 0.24743432 |
| TCGA-BH-A0HU | 392 | 0 | 0.108512006 |
| TCGA-E9-A54X | 375 | 0 | 0.323581738 |
| TCGA-AO-A03U | 1793 | 1 | 0.221575342 |
| TCGA-BH-A0WA | 701 | 0 | 0.122492994 |
| TCGA-AC-A4ZE | 890 | 0 | 0.181048127 |
| TCGA-BH-A18S | 2009 | 1 | 0.355562093 |
| TCGA-GM-A2DH | 2193 | 0 | 0.087557153 |
| TCGA-C8-A26Y | 0 | 0 | 0.112443957 |
| TCGA-A8-A09B | 365 | 0 | 0.295045114 |
| TCGA-EW-A423 | 309 | 0 | 0.312629514 |
| TCGA-AC-A2FE | 2240 | 0 | 0.098818182 |
| TCGA-AR-A24X | 2548 | 0 | 0.090063564 |
| TCGA-GM-A4E0 | 2191 | 0 | 0.22699883 |
| TCGA-A2-A0EU | 1043 | 0 | 0.078843842 |
| TCGA-A7-A0CJ | 931 | 0 | 0.113588717 |
| TCGA-GI-A2C9 | 3342 | 0 | 0.260844219 |
| TCGA-BH-A0DI | 912 | 0 | 0.117928751 |
| TCGA-A1-A0SN | 1196 | 0 | 0.15999701 |
| TCGA-BH-A18J | 612 | 1 | 0.25660345 |
| TCGA-BH-A1EY | 538 | 1 | 0.15273991 |
| TCGA-D8-A27V | 381 | 0 | 0.164169126 |
| TCGA-5L-AAT1 | 1471 | 0 | 0.122880434 |
| TCGA-E2-A14Y | 2109 | 0 | 0.111965911 |
| TCGA-AO-A125 | 3456 | 0 | 0.033555287 |
| TCGA-EW-A424 | 715 | 0 | 0.104433852 |
| TCGA-AO-A12H | 1234 | 0 | 0.340645211 |
| TCGA-BH-A0B5 | 2136 | 0 | 0.11907169 |
| TCGA-BH-A0C3 | 2709 | 0 | 0.109828954 |
| TCGA-E9-A1NC | 1203 | 0 | 0.059484272 |
| TCGA-AC-A2FG | 1853 | 0 | 0.13661362 |
| TCGA-BH-A204 | 2534 | 1 | 0.547296476 |
| TCGA-D8-A1XV | 461 | 0 | 0.195633507 |
| TCGA-BH-A8FY | 295 | 1 | 0.268139364 |
| TCGA-A2-A0YH | 659 | 0 | 0.288314272 |
| TCGA-A8-A09D | 1522 | 0 | 0.266960395 |
| TCGA-BH-A0AW | 622 | 0 | 0.108486073 |
| TCGA-AR-A0TX | 1972 | 0 | 0.195451057 |
| TCGA-AC-A2FM | 792 | 1 | 0.211534205 |
| TCGA-EW-A2FW | 672 | 0 | 0.154797176 |
| TCGA-A2-A0D2 | 1027 | 0 | 0.103278322 |
| TCGA-BH-A0GY | 923 | 0 | 0.09156586 |
| TCGA-OL-A66P | 428 | 0 | 0.098129612 |
| TCGA-E2-A1L7 | 1836 | 0 | 0.119558194 |
| TCGA-D8-A1JF | 366 | 0 | 0.180415659 |
| TCGA-D8-A1J8 | 431 | 0 | 0.15373015 |
| TCGA-BH-A0DV | 2064 | 0 | 0.225189108 |
| TCGA-S3-AA14 | 455 | 0 | 0.160283404 |
| TCGA-A8-A08R | 30 | 0 | 0.11793468 |
| TCGA-C8-A1HI | 343 | 0 | 0.241503386 |
| TCGA-D8-A27I | 439 | 0 | 0.154665796 |
| TCGA-BH-A203 | 1174 | 1 | 0.164223527 |
| TCGA-E9-A229 | 1148 | 0 | 0.250368333 |
| TCGA-BH-A18N | 468 | 1 | 0.359513679 |
| TCGA-A1-A0SJ | 416 | 0 | 0.135087422 |
| TCGA-E2-A1LK | 266 | 1 | 0.14468108 |
| TCGA-AN-A046 | 10 | 0 | 0.116486813 |
| TCGA-OL-A5DA | 1783 | 0 | 0.032651457 |
| TCGA-BH-A18V | 1556 | 1 | 0.141348798 |
| TCGA-D8-A1X9 | 727 | 0 | 0.201634773 |
| TCGA-C8-A134 | 383 | 0 | 0.087103908 |
| TCGA-EW-A1P3 | 1611 | 0 | 0.139483441 |
| TCGA-V7-A7HQ | 2033 | 0 | 0.18517443 |
| TCGA-C8-A1HJ | 5 | 0 | 0.085400053 |
| TCGA-C8-A275 | 1 | 0 | 0.10920553 |
| TCGA-AR-A2LL | 1719 | 0 | 0.16488389 |
| TCGA-AC-A62X | 417 | 0 | 0 |
| TCGA-E9-A5FK | 447 | 0 | 0.05469667 |
| TCGA-B6-A1KF | 3088 | 0 | 0.149315502 |
| TCGA-AC-A6NO | 51 | 0 | 0.108419932 |
| TCGA-A2-A4RW | 222 | 0 | 0.1309972 |
| TCGA-A7-A3IY | 345 | 0 | 0.173074349 |
| TCGA-BH-A0H3 | 1928 | 0 | 0.154406252 |
| TCGA-A1-A0SI | 635 | 0 | 0.111161616 |
| TCGA-AC-A23H | 0 | 0 | 0.09679945 |
| TCGA-E9-A24A | 747 | 0 | 0.382894019 |
| TCGA-OL-A66O | 528 | 0 | 0.151526768 |
| TCGA-B6-A0IN | 2573 | 1 | 0.084577926 |
| TCGA-EW-A1P4 | 907 | 0 | 0.056764237 |
| TCGA-XX-A89A | 488 | 0 | 0.150211605 |
| TCGA-A2-A0SW | 1365 | 1 | 0.135232188 |
| TCGA-C8-A133 | 0 | 0 | 0.451463732 |
| TCGA-B6-A0I9 | 362 | 1 | 0.13718296 |
| TCGA-E9-A1RG | 647 | 0 | 0.209910836 |
| TCGA-A8-A0AD | 1157 | 0 | 0.290723699 |
| TCGA-E2-A15T | 1563 | 0 | 0.10976166 |
| TCGA-LL-A441 | 996 | 0 | 0.052810185 |
| TCGA-A8-A08C | 881 | 0 | 0.220397488 |
| TCGA-LL-A9Q3 | 532 | 0 | 0.185500402 |
| TCGA-A7-A3J0 | 313 | 0 | 0.027320899 |
| TCGA-AO-A03L | 2442 | 0 | 0.074055213 |
| TCGA-BH-A0HL | 72 | 0 | 0.274351982 |
| TCGA-D8-A147 | 584 | 0 | 0.101272379 |
| TCGA-D8-A4Z1 | 659 | 0 | 0.125336225 |
| TCGA-B6-A2IU | 5176 | 0 | 0.147668019 |
| TCGA-AR-A24H | 4776 | 0 | 0.149797588 |
| TCGA-5T-A9QA | 303 | 0 | 0.364040758 |
| TCGA-E2-A1LL | 1309 | 0 | 0.19282087 |
| TCGA-E9-A5FL | 24 | 0 | 0.437332458 |
| TCGA-AR-A2LK | 1649 | 1 | 0.296639628 |
| TCGA-BH-A0HQ | 1121 | 0 | 0.074288559 |
| TCGA-OK-A5Q2 | 64 | 0 | 0.063365446 |
| TCGA-AN-A0FN | 218 | 0 | 0.08821405 |
| TCGA-E2-A1IO | 1855 | 0 | 0.063548318 |
| TCGA-E2-A574 | 808 | 0 | 0.059928274 |
| TCGA-EW-A1J6 | 875 | 0 | 0.185307595 |
| TCGA-E2-A15R | 1732 | 0 | 0.350080355 |
| TCGA-A7-A5ZX | 336 | 0 | 0.199493942 |
| TCGA-B6-A0X0 | 3945 | 1 | 0.095451215 |
| TCGA-E2-A10A | 1229 | 0 | 0.195738792 |
| TCGA-A2-A0EQ | 2426 | 0 | 0.059810066 |
| TCGA-A7-A26E | 954 | 0 | 0.186226192 |
| TCGA-A2-A25F | 322 | 0 | 0.108688787 |
| TCGA-S3-A6ZH | 515 | 0 | 0.123228428 |
| TCGA-A8-A09G | 0 | 0 | 0.177910151 |
| TCGA-B6-A0WZ | 6292 | 0 | 0.197581729 |
| TCGA-AC-A62Y | 530 | 0 | 0.200500007 |
| TCGA-C8-A12O | 385 | 0 | 0.159299422 |
| TCGA-BH-A0C7 | 2767 | 0 | 0.126231215 |
| TCGA-A8-A09M | 1006 | 0 | 0.20281241 |
| TCGA-AR-A2LQ | 895 | 0 | 0.198537972 |
| TCGA-S3-AA17 | 424 | 0 | 0.100608586 |
| TCGA-BH-A0HK | 178 | 0 | 0.168552772 |
| TCGA-E2-A573 | 1062 | 0 | 0.105526595 |
| TCGA-A8-A09W | 30 | 0 | 0.25273458 |
| TCGA-E2-A15C | 694 | 0 | 0.091384065 |
| TCGA-E2-A1IE | 2362 | 0 | 0.206821587 |
| TCGA-E9-A1RD | 34 | 0 | 0.286087757 |
| TCGA-D8-A143 | 431 | 0 | 0.171816001 |
| TCGA-AO-A128 | 3248 | 0 | 0.161290333 |
| TCGA-AO-A12F | 1842 | 0 | 0.321066414 |
| TCGA-A8-A08T | 3409 | 1 | 0.17137798 |
| TCGA-C8-A137 | 379 | 0 | 0.164366209 |
| TCGA-BH-A1FJ | 1927 | 1 | 0.285026565 |
| TCGA-OL-A6VR | 1220 | 0 | 0.183070263 |
| TCGA-OL-A5RU | 1219 | 0 | 0.05958148 |
| TCGA-BH-A0BF | 1324 | 1 | 0.155494978 |
| TCGA-MS-A51U | 681 | 0 | 0.053163862 |
| TCGA-BH-A0B8 | 1569 | 0 | 0.198888922 |
| TCGA-A2-A0CW | 3283 | 0 | 0.173341039 |
| TCGA-LL-A5YM | 394 | 0 | 0.250888632 |
| TCGA-BH-A0H7 | 702 | 0 | 0.121629085 |
| TCGA-A8-A07G | 577 | 0 | 0.164950291 |
| TCGA-E9-A3QA | 918 | 0 | 0.052368376 |
| TCGA-A2-A0CM | 754 | 1 | 0.084122855 |
| TCGA-AO-A0J9 | 1613 | 0 | 0.132410257 |
| TCGA-C8-A12Z | 382 | 0 | 0.103509241 |
| TCGA-AN-A0FV | 10 | 0 | 0.228952483 |
| TCGA-AR-A0TY | 1699 | 1 | 0.21268126 |
| TCGA-A8-A07W | 304 | 0 | 0.209665633 |
| TCGA-B6-A0RH | 6456 | 1 | 0.08550544 |
| TCGA-AR-A1AW | 1935 | 0 | 0.098312453 |
| TCGA-C8-A12P | 358 | 0 | 0.210642183 |
| TCGA-HN-A2NL | 79 | 0 | 0.125539482 |
| TCGA-EW-A1P7 | 915 | 0 | 0.042403655 |
| TCGA-GM-A2DF | 2155 | 0 | 0.112068996 |
| TCGA-AR-A1AM | 1903 | 0 | 0.113712256 |
| TCGA-EW-A6SC | 952 | 0 | 0.154220271 |
| TCGA-A8-A083 | 0 | 0 | 0.319098392 |
| TCGA-E9-A249 | 217 | 0 | 0.136729729 |
| TCGA-BH-A1FN | 2192 | 1 | 0.102577941 |
| TCGA-A2-A0T7 | 631 | 0 | 0.200403357 |
| TCGA-A8-A06Z | 31 | 0 | 0.378683976 |
| TCGA-AO-A0JJ | 1887 | 0 | 0.151414436 |
| TCGA-E2-A14X | 972 | 0 | 0.074949637 |
| TCGA-A8-A06P | 396 | 0 | 0.261034083 |
| TCGA-LL-A442 | 889 | 0 | 0.624054539 |
| TCGA-E9-A1RB | 976 | 1 | 0.157324824 |
| TCGA-BH-A1EX | 1508 | 1 | 0.20999979 |
| TCGA-D8-A1XA | 839 | 0 | 0.144391735 |
| TCGA-E2-A1IG | 2140 | 0 | 0.235013383 |
| TCGA-A8-A076 | 1642 | 0 | 0.215166568 |
| TCGA-AN-A04D | 52 | 0 | 0.164044852 |
| TCGA-AC-A3OD | 451 | 0 | 0.128675867 |
| TCGA-A2-A0YF | 1535 | 0 | 0.220206182 |
| TCGA-AR-A1AP | 2856 | 0 | 0.250762364 |
| TCGA-BH-A0B1 | 1148 | 0 | 0.123213829 |
| TCGA-C8-A12W | 385 | 0 | 0.173287105 |
| TCGA-A8-A07P | 334 | 0 | 0.264598884 |
| TCGA-C8-A12M | 358 | 0 | 0.123427809 |
| TCGA-C8-A26X | 376 | 0 | 0.140669547 |
| TCGA-A2-A0CZ | 1616 | 0 | 0.069407581 |
| TCGA-OL-A6VQ | 600 | 0 | 0.078861775 |
| TCGA-A8-A08L | 30 | 1 | 0.129821574 |
| TCGA-A8-A084 | 458 | 0 | 0.135969512 |
| TCGA-A7-A0DC | 906 | 0 | 0.230761666 |
| TCGA-A2-A3XT | 2525 | 0 | 0.06339926 |
| TCGA-BH-A1F0 | 785 | 1 | 0.199432508 |
| TCGA-A8-A07E | 608 | 0 | 0.152527982 |
| TCGA-D8-A1JH | 426 | 0 | 0.146575062 |
| TCGA-E9-A54Y | 384 | 0 | 0.083981194 |
| TCGA-A2-A04N | 4354 | 0 | 0.172753276 |
| TCGA-A2-A0CP | 2813 | 0 | 0.163390632 |
| TCGA-EW-A1PC | 187 | 0 | 0.180153861 |
| TCGA-LL-A5YP | 450 | 0 | 0.225372209 |
| TCGA-A8-A0A6 | 640 | 0 | 0.145516399 |
| TCGA-XX-A899 | 467 | 0 | 0.11591234 |
| TCGA-A8-A07Z | 1371 | 0 | 0.422843051 |
| TCGA-B6-A0IA | 8391 | 0 | 0.086616138 |
| TCGA-EW-A1OX | 911 | 0 | 0.095334799 |
| TCGA-AR-A2LR | 846 | 0 | 0.374377501 |
| TCGA-A7-A26G | 722 | 0 | 0.11227519 |
| TCGA-B6-A40B | 3152 | 0 | 0.087865661 |
| TCGA-A2-A04V | 1920 | 1 | 0.103689766 |
| TCGA-AN-A0FJ | 242 | 0 | 0.055474861 |
| TCGA-LL-A8F5 | 596 | 0 | 0.104434623 |
| TCGA-BH-A0E6 | 293 | 0 | 0.071260707 |
| TCGA-A8-A096 | 0 | 0 | 0.202867919 |
| TCGA-AR-A254 | 2213 | 0 | 0.225552854 |
| TCGA-E2-A1AZ | 2329 | 0 | 0.06606116 |
| TCGA-A8-A06O | 396 | 0 | 0.262417651 |
| TCGA-AC-A8OS | 70 | 0 | 0.138527354 |
| TCGA-E9-A1N4 | 1000 | 0 | 0.128678367 |
| TCGA-E2-A15K | 275 | 0 | 0.091197827 |
| TCGA-BH-A6R8 | 293 | 0 | 0.229088696 |
| TCGA-A7-A4SA | 454 | 0 | 0.089884574 |
| TCGA-A2-A0D1 | 1051 | 0 | 0.122480244 |
| TCGA-BH-A0EE | 943 | 0 | 0.082227526 |
| TCGA-A8-A07O | 304 | 0 | 0.055659039 |
| TCGA-AR-A1AO | 2618 | 0 | 0.100167264 |
| TCGA-A2-A0ER | 2263 | 0 | 0.187885003 |
| TCGA-B6-A0WW | 558 | 1 | 0.161489948 |
| TCGA-E2-A1B5 | 984 | 0 | 0.048074108 |
| TCGA-E9-A22A | 1189 | 0 | 0.154605505 |
| TCGA-LL-A740 | 441 | 0 | 0.161512546 |
| TCGA-A7-A6VW | 285 | 0 | 0.106101736 |
| TCGA-C8-A278 | 297 | 0 | 0.166577172 |
| TCGA-AO-A0JI | 1528 | 0 | 0.033168828 |
| TCGA-AC-A6IW | 413 | 0 | 0.165996083 |
| TCGA-AR-A5QQ | 322 | 1 | 0.136084998 |
| TCGA-A2-A0CO | 1468 | 0 | 0.067723356 |
| TCGA-D8-A13Z | 635 | 0 | 0.192596049 |
| TCGA-AC-A2FB | 1234 | 0 | 0.057713469 |
| TCGA-E9-A1N3 | 1059 | 0 | 0.34283267 |
| TCGA-A8-A09Z | 0 | 0 | 0.175712747 |
| TCGA-AO-A03R | 2091 | 0 | 0.09018643 |
| TCGA-AQ-A04L | 3957 | 0 | 0.232723117 |
| TCGA-A2-A3XU | 912 | 1 | 0.141449451 |
| TCGA-AN-A0FS | 210 | 0 | 0.308209596 |
| TCGA-A8-A09E | 1492 | 0 | 0.154426741 |
| TCGA-LL-A5YO | 440 | 0 | 0.063833308 |
| TCGA-A8-A0A1 | 365 | 0 | 0.10343161 |
| TCGA-A2-A3XS | 1032 | 1 | 0.10408253 |
| TCGA-A2-A0YM | 965 | 0 | 0.080122194 |
| TCGA-A1-A0SK | 967 | 1 | 0 |
| TCGA-E9-A3HO | 793 | 0 | 0.07900146 |
| TCGA-D8-A1XC | 377 | 1 | 0.120008659 |
| TCGA-AO-A126 | 3307 | 0 | 0.216754044 |
| TCGA-BH-A18R | 1142 | 1 | 0.148799261 |
| TCGA-A2-A0YG | 666 | 0 | 0.181009493 |
| TCGA-AN-A0XW | 170 | 0 | 0.122813062 |
| TCGA-EW-A1OW | 694 | 0 | 0.078568415 |
| TCGA-E9-A5UP | 438 | 0 | 0 |
| TCGA-EW-A6SA | 510 | 0 | 0.184016517 |
| TCGA-E2-A1LI | 3121 | 0 | 0.07950679 |
| TCGA-BH-A0B6 | 2483 | 0 | 0.068119607 |
| TCGA-E9-A244 | 21 | 0 | 0.11437959 |
| TCGA-AQ-A54O | 1001 | 0 | 0.103906519 |
| TCGA-B6-A0IC | 0 | 1 | 0.200469419 |
| TCGA-EW-A1PA | 575 | 0 | 0.068864474 |
| TCGA-BH-A0BP | 2296 | 1 | 0.141471325 |
| TCGA-PE-A5DE | 2645 | 0 | 0.059905397 |
| TCGA-E2-A2P5 | 821 | 1 | 0.035787688 |
| TCGA-D8-A1Y1 | 302 | 1 | 0.344547316 |
| TCGA-BH-A1FL | 1673 | 1 | 0.138101689 |
| TCGA-E2-A1IF | 1138 | 0 | 0.118016689 |
| TCGA-AR-A0U2 | 2551 | 1 | 0.164675481 |
| TCGA-A2-A4S1 | 820 | 0 | 0.586487279 |
| TCGA-AC-A6IX | 373 | 0 | 0.219650322 |
| TCGA-A7-A0DA | 1085 | 0 | 0.196670744 |
| TCGA-A7-A6VX | 317 | 0 | 0.22825527 |
| TCGA-E9-A243 | 612 | 0 | 0.112047761 |
| TCGA-A1-A0SQ | 554 | 0 | 0.1809936 |
| TCGA-BH-A0HA | 1611 | 0 | 0.066215185 |
| TCGA-B6-A0WX | 639 | 1 | 0.061375465 |
| TCGA-OL-A5RV | 1062 | 0 | 0.135658778 |
| TCGA-BH-A0BZ | 2255 | 0 | 0.129827662 |
| TCGA-AO-A12E | 2142 | 0 | 0.201459444 |
| TCGA-A7-A3IZ | 322 | 0 | 0.24835543 |
| TCGA-GM-A2DO | 2596 | 0 | 0.041570864 |
| TCGA-3C-AAAU | 3928 | 0 | 0.256778612 |
| TCGA-E9-A1N9 | 1101 | 0 | 0.108407649 |
| TCGA-AC-A23G | 2248 | 0 | 0.091719624 |
| TCGA-E2-A107 | 1047 | 0 | 0.130921768 |
| TCGA-AC-A3QQ | 734 | 0 | 0.089460381 |
| TCGA-E2-A1LS | 1604 | 0 | 0.204983113 |
| TCGA-AN-A0FT | 214 | 0 | 0.197957107 |
| TCGA-S3-AA0Z | 511 | 0 | 0.112097845 |
| TCGA-E2-A14W | 974 | 0 | 0.308934864 |
| TCGA-AR-A250 | 2274 | 0 | 0.173220625 |
| TCGA-A2-A25E | 3204 | 0 | 0.175612978 |
| TCGA-BH-A1EW | 1694 | 1 | 0.082049041 |
| TCGA-D8-A3Z6 | 563 | 0 | 0.135170017 |
| TCGA-E9-A5UO | 420 | 0 | 0.401335753 |
| TCGA-D8-A1JB | 1688 | 0 | 0.096883114 |
| TCGA-D8-A1XR | 482 | 0 | 0.233896377 |
| TCGA-BH-A0BO | 2197 | 0 | 0.119836574 |
| TCGA-BH-A8FZ | 574 | 0 | 0.089194389 |
| TCGA-A7-A425 | 447 | 0 | 0.378151224 |
| TCGA-A8-A091 | 1004 | 0 | 0.246112281 |
| TCGA-D8-A27K | 1461 | 0 | 0.081578469 |
| TCGA-A2-A3KD | 1206 | 0 | 0.147202915 |
| TCGA-BH-A0E1 | 477 | 0 | 0.13570195 |
| TCGA-A7-A26F | 738 | 0 | 0.182959334 |
| TCGA-C8-A1HK | 366 | 0 | 0.089788935 |
| TCGA-C8-A26W | 381 | 0 | 0.157119653 |
| TCGA-E9-A1R5 | 92 | 0 | 0.176761064 |
| TCGA-BH-A0GZ | 328 | 0 | 0.147530894 |
| TCGA-AO-A1KR | 2513 | 0 | 0.063417664 |
| TCGA-E9-A1RH | 1052 | 0 | 0.088575639 |
| TCGA-A7-A4SC | 446 | 0 | 0.132152861 |
| TCGA-AC-A3W5 | 504 | 0 | 0.12350453 |
| TCGA-C8-A12X | 385 | 0 | 0.30841475 |
| TCGA-E2-A10C | 1220 | 0 | 0.121061291 |
| TCGA-AO-A1KQ | 1882 | 0 | 0.089742031 |
| TCGA-B6-A0X4 | 860 | 1 | 0.332194796 |
| TCGA-D8-A1JD | 552 | 0 | 0.240710029 |
| TCGA-E2-A14Z | 563 | 1 | 0.139688205 |
| TCGA-B6-A402 | 2281 | 0 | 0.092018223 |
| TCGA-AC-A7VC | 1 | 0 | 0.140818049 |
| TCGA-AN-A0XO | 375 | 0 | 0.045067298 |
| TCGA-BH-A0W4 | 759 | 0 | 0.093806957 |
| TCGA-BH-A0DQ | 98 | 0 | 0.120273041 |
| TCGA-B6-A0IQ | 4285 | 0 | 0.060752678 |
| TCGA-AQ-A04J | 819 | 0 | 0.063510836 |
| TCGA-E2-A570 | 931 | 0 | 0.18236187 |
| TCGA-LL-A6FP | 0 | 0 | 0.282688952 |
| TCGA-A2-A3XV | 699 | 0 | 0.265984234 |
| TCGA-D8-A1XK | 441 | 0 | 0.09846486 |
| TCGA-AC-A23E | 698 | 0 | 0.39075501 |
| TCGA-BH-A1FU | 1688 | 1 | 0.076109994 |
| TCGA-E2-A14P | 1246 | 0 | 0.104030242 |
| TCGA-A8-A06X | 943 | 1 | 0.209801066 |
| TCGA-E2-A1L9 | 598 | 0 | 0.16084517 |
| TCGA-BH-A0W3 | 728 | 0 | 0.297218327 |
| TCGA-A2-A04U | 2654 | 0 | 0.175843426 |
| TCGA-D8-A73U | 492 | 0 | 0.125368742 |
| TCGA-D8-A1XQ | 499 | 0 | 0.215653418 |
| TCGA-EW-A1J1 | 575 | 0 | 0.172933075 |
| TCGA-AR-A1AX | 1890 | 0 | 0.076093664 |
| TCGA-BH-A0AY | 777 | 0 | 0.184993672 |
| TCGA-A8-A08I | 365 | 0 | 0.104437217 |
| TCGA-AN-A0FL | 231 | 0 | 0.180003315 |
| TCGA-E9-A227 | 975 | 0 | 0.182601861 |
| TCGA-D8-A27R | 307 | 0 | 0.230585917 |
| TCGA-C8-A26Z | 470 | 0 | 0.23407579 |
| TCGA-BH-A0DK | 423 | 0 | 0.102726973 |
| TCGA-B6-A0IK | 571 | 1 | 0.075372014 |
| TCGA-A2-A0CX | 1728 | 0 | 0.209712963 |
| TCGA-E9-A1NI | 300 | 0 | 0.260038858 |
| TCGA-A2-A0SY | 1347 | 0 | 0.118050911 |
| TCGA-BH-A8G0 | 662 | 0 | 0.100841755 |
| TCGA-LL-A50Y | 762 | 0 | 0.12785701 |
| TCGA-A7-A5ZW | 326 | 0 | 0.165855375 |
| TCGA-D8-A1X7 | 509 | 0 | 0.268753718 |
| TCGA-AN-A0XP | 9 | 0 | 0.399903594 |
| TCGA-OL-A66H | 812 | 0 | 0.363409538 |
| TCGA-A2-A0YE | 554 | 0 | 0.108674385 |
| TCGA-EW-A1OZ | 1229 | 0 | 0.217330507 |
| TCGA-A8-A08J | 1127 | 1 | 0.294878185 |
| TCGA-E2-A1BD | 1133 | 0 | 0.184422218 |
| TCGA-GM-A2DM | 3226 | 0 | 0.238310243 |
| TCGA-BH-A18K | 2763 | 1 | 0.219488814 |
| TCGA-AO-A0J3 | 651 | 0 | 0.251176654 |
| TCGA-A8-A07F | 577 | 0 | 0.170763234 |
| TCGA-E2-A15A | 710 | 0 | 0.147281852 |
| TCGA-E9-A1R2 | 1063 | 0 | 0.078477767 |
| TCGA-BH-A1EO | 2798 | 1 | 0.165841594 |
| TCGA-E2-A14O | 1359 | 0 | 0.061054123 |
| TCGA-LL-A73Z | 227 | 1 | 0.122453722 |
| TCGA-A8-A08S | 1004 | 0 | 0.155484686 |
| TCGA-A8-A09X | 426 | 1 | 0.149470942 |
| TCGA-AO-A12G | 1639 | 0 | 0.13617883 |
| TCGA-BH-A0BG | 1871 | 0 | 0.069521291 |
| TCGA-BH-A18Q | 1692 | 1 | 0.137449452 |
| TCGA-AC-A2BK | 2222 | 0 | 0.233872428 |
| TCGA-A2-A04T | 2246 | 0 | 0.116852139 |
| TCGA-LD-A9QF | 323 | 0 | 0.070655398 |
| TCGA-A2-A4RY | 648 | 0 | 0.10899343 |
| TCGA-BH-A0BW | 2371 | 0 | 0.144526387 |
| TCGA-AO-A0J4 | 1587 | 0 | 0.073312277 |
| TCGA-AO-A0JL | 1683 | 0 | 0.123026581 |
| TCGA-D8-A140 | 403 | 0 | 0.13538296 |
| TCGA-BH-A0BM | 1876 | 0 | 0.135913837 |
| TCGA-A2-A04X | 1686 | 0 | 0.117211726 |
| TCGA-D8-A73X | 368 | 0 | 0.187612518 |
| TCGA-GM-A2DI | 2590 | 0 | 0.085445257 |
| TCGA-EW-A1OV | 789 | 0 | 0.087216985 |
| TCGA-E2-A1LE | 879 | 1 | 0.131258115 |
| TCGA-A8-A07U | 760 | 0 | 0.083235552 |
| TCGA-AN-A0XV | 162 | 0 | 0.115711821 |
| TCGA-AR-A1AU | 2318 | 0 | 0.156878014 |
| TCGA-A8-A09T | 579 | 0 | 0.344068951 |
| TCGA-E9-A1NG | 786 | 1 | 0.460643924 |
| TCGA-D8-A1X5 | 565 | 0 | 0.239791649 |
| TCGA-AC-A3EH | 197 | 1 | 0.20633468 |
| TCGA-E9-A6HE | 468 | 0 | 0.177215661 |
| TCGA-A2-A0CU | 158 | 1 | 0.172574731 |
| TCGA-B6-A0X1 | 7455 | 1 | 0.103152479 |
| TCGA-EW-A1J3 | 504 | 0 | 0.169924292 |
| TCGA-A7-A3RF | 408 | 0 | 0.213803767 |
| TCGA-BH-A1EN | 2127 | 1 | 0.319157239 |
| TCGA-E2-A14N | 1434 | 0 | 0.068842924 |
| TCGA-E2-A56Z | 252 | 0 | 0.034641224 |
| TCGA-BH-A6R9 | 160 | 0 | 0.184834995 |
| TCGA-E2-A1L6 | 1648 | 0 | 0.118757809 |
| TCGA-A7-A13H | 740 | 0 | 0.062278634 |
| TCGA-OL-A5RW | 1106 | 0 | 0.14201487 |
| TCGA-AC-A3BB | 987 | 0 | 0.094083613 |
| TCGA-A7-A56D | 448 | 0 | 0.188514655 |
| TCGA-E2-A576 | 1043 | 0 | 0.068565969 |
| TCGA-B6-A40C | 2164 | 0 | 0.261249954 |
| TCGA-B6-A0IH | 2965 | 1 | 0.087764443 |
| TCGA-BH-A0DH | 1156 | 0 | 0.125122712 |
| TCGA-BH-A2L8 | 612 | 0 | 0.045146779 |
| TCGA-D8-A1JA | 502 | 0 | 0.2701934 |
| TCGA-B6-A0I5 | 8556 | 0 | 0.11138597 |
| TCGA-C8-A3M8 | 394 | 0 | 0.175833093 |
| TCGA-3C-AALI | 4005 | 0 | 0.056670324 |
| TCGA-A8-A06U | 883 | 1 | 0.260892954 |
| TCGA-AR-A1AT | 1272 | 1 | 0.167577701 |
| TCGA-D8-A146 | 643 | 0 | 0.116759146 |
| TCGA-EW-A1PB | 608 | 0 | 0.196005503 |
| TCGA-BH-A0BJ | 660 | 0 | 0.124843631 |
| TCGA-OL-A66K | 1275 | 1 | 0.177695375 |
| TCGA-BH-A1F8 | 1 | 1 | 0.202504513 |
| TCGA-A2-A3XZ | 1165 | 0 | 0.147770676 |
| TCGA-E2-A1IL | 118 | 0 | 0.174706078 |
| TCGA-A2-A0CT | 2289 | 0 | 0.173615408 |
| TCGA-BH-A18H | 652 | 0 | 0.045012764 |
| TCGA-A7-A0DB | 1007 | 0 | 0.147772351 |
| TCGA-EW-A2FR | 1673 | 0 | 0.163329629 |
| TCGA-AQ-A1H3 | 989 | 0 | 0.09737354 |
| TCGA-BH-A0HB | 806 | 0 | 0.15168568 |
| TCGA-BH-A1EV | 365 | 1 | 0.141619423 |
| TCGA-E2-A14V | 1042 | 0 | 0.098214698 |
| TCGA-3C-AALJ | 1302 | 0 | 0.116827128 |
| TCGA-GM-A3NY | 1162 | 0 | 0.037893681 |
| TCGA-E9-A22H | 630 | 0 | 0.174779084 |
| TCGA-OL-A5D8 | 973 | 0 | 0.131490855 |
| TCGA-C8-A26V | 616 | 0 | 0.154741176 |
| TCGA-E2-A15D | 526 | 0 | 0.132969532 |
| TCGA-PL-A8LY | 8 | 0 | 0.187716149 |
| TCGA-A7-A0CH | 1079 | 0 | 0.217269704 |
| TCGA-C8-A12L | 363 | 0 | 0.123920728 |
| TCGA-E9-A1RC | 1224 | 0 | 0.024001476 |
| TCGA-AN-A0XN | 10 | 0 | 0.215786168 |
| TCGA-A8-A06T | 1614 | 0 | 0.136273071 |
| TCGA-A8-A08G | 607 | 0 | 0.219221849 |
| TCGA-E9-A248 | 59 | 0 | 0.095575935 |
| TCGA-EW-A6SB | 760 | 0 | 0.045961487 |
| TCGA-A2-A3XW | 1505 | 0 | 0.136697817 |
| TCGA-A2-A4S3 | 666 | 0 | 0.07662527 |
| TCGA-GM-A2D9 | 1812 | 1 | 0.200697331 |
| TCGA-A1-A0SH | 1437 | 0 | 0.176820963 |
| TCGA-A8-A086 | 396 | 0 | 0.262616108 |
| TCGA-AC-A2FK | 2650 | 0 | 0.084386415 |
| TCGA-C8-A12T | 0 | 0 | 0.175542409 |
| TCGA-B6-A0I2 | 4361 | 0 | 0.063437549 |
| TCGA-AO-A0J8 | 680 | 0 | 0.044919232 |
| TCGA-AO-A0JF | 1980 | 0 | 0.164377074 |
| TCGA-E2-A105 | 1308 | 0 | 0.135261728 |
| TCGA-BH-A5J0 | 715 | 0 | 0.152062064 |
| TCGA-AN-A0XS | 10 | 0 | 0.083829638 |
| TCGA-A8-A0A4 | 396 | 0 | 0.247994435 |
| TCGA-EW-A1PD | 424 | 0 | 0.116115356 |
| TCGA-D8-A1Y3 | 430 | 0 | 0.434377801 |
| TCGA-LL-A5YL | 519 | 0 | 0.163819841 |
| TCGA-BH-A0B0 | 2477 | 0 | 0.072699514 |
| TCGA-AC-A3W7 | 471 | 0 | 0.185774867 |
| TCGA-EW-A6SD | 1010 | 0 | 0.130499689 |
| TCGA-AN-A0FX | 10 | 0 | 0.083256557 |
| TCGA-BH-A0B9 | 1572 | 0 | 0.076378736 |
| TCGA-AO-A129 | 3286 | 0 | 0.076121943 |
| TCGA-E9-A1QZ | 755 | 0 | 0.085958999 |
| TCGA-E9-A1R7 | 1130 | 0 | 0.041624769 |
| TCGA-A2-A0CL | 3015 | 0 | 0.033674443 |
| TCGA-A8-A08Z | 1217 | 0 | 0.17969083 |
| TCGA-A8-A08P | 943 | 0 | 0.158922012 |
| TCGA-AN-A04C | 54 | 0 | 0.243481843 |
| TCGA-AQ-A7U7 | 584 | 1 | 0.100000158 |
| TCGA-A2-A0YJ | 566 | 0 | 0.13909617 |
| TCGA-GM-A2DN | 3091 | 0 | 0.05857941 |
| TCGA-AR-A1AL | 2971 | 0 | 0.107510401 |
| TCGA-D8-A1XY | 503 | 0 | 0.295589724 |
| TCGA-A8-A07L | 975 | 0 | 0.218074092 |
| TCGA-Z7-A8R6 | 3256 | 0 | 0.112410157 |
| TCGA-AC-A5XU | 455 | 0 | 0.122669125 |
| TCGA-BH-A1ES | 3462 | 1 | 0.260445737 |
| TCGA-E2-A1LG | 1523 | 0 | 0.08762859 |
| TCGA-E9-A1NE | 1088 | 0 | 0.065239679 |
| TCGA-AN-A0FF | 172 | 0 | 0.249357246 |
| TCGA-B6-A0WT | 5739 | 0 | 0.147056598 |
| TCGA-A8-A093 | 546 | 0 | 0.099860585 |
| TCGA-AQ-A54N | 78 | 0 | 0.164269511 |
| TCGA-C8-A12U | 385 | 0 | 0.104434479 |
| TCGA-A2-A0YI | 1505 | 0 | 0.163628839 |
| TCGA-A2-A259 | 1596 | 0 | 0.097214281 |
| TCGA-A7-A5ZV | 368 | 0 | 0.156242838 |
| TCGA-A2-A0D0 | 2048 | 0 | 0.098327215 |
| TCGA-OL-A5RZ | 679 | 0 | 0.186823065 |
| TCGA-BH-A0BV | 1519 | 0 | 0.159335335 |
| TCGA-AC-A3HN | 496 | 0 | 0.117342524 |
| TCGA-A8-A094 | 0 | 0 | 0.166357684 |
| TCGA-A8-A08O | 943 | 0 | 0.082623406 |
| TCGA-AR-A256 | 2854 | 1 | 0.110077473 |
| TCGA-E2-A1IU | 337 | 0 | 0.128435075 |
| TCGA-E9-A1N6 | 678 | 1 | 0.248127734 |
| TCGA-D8-A27H | 397 | 0 | 0.172724795 |
| TCGA-BH-A0DD | 2486 | 0 | 0.121350979 |
| TCGA-A8-A0A9 | 822 | 0 | 0.139398733 |
| TCGA-LL-A5YN | 447 | 0 | 0.062373031 |
| TCGA-A2-A0EY | 1925 | 0 | 0.120762143 |
| TCGA-AC-A3YJ | 754 | 0 | 0.387887092 |
| TCGA-A7-A26I | 661 | 0 | 0.325386554 |
| TCGA-A2-A04P | 548 | 1 | 0.077067897 |
| TCGA-BH-A1F6 | 2965 | 1 | 0.197965086 |
| TCGA-C8-A8HQ | 380 | 0 | 0.101003849 |
| TCGA-BH-A0HY | 1545 | 0 | 0.172858378 |
| TCGA-AN-A0G0 | 16 | 0 | 0.156340176 |
| TCGA-BH-A0B3 | 1203 | 0 | 0.10786304 |
| TCGA-AR-A1AN | 2920 | 0 | 0.087937411 |
| TCGA-A7-A13D | 965 | 0 | 0.03316567 |
| TCGA-GM-A2DL | 3519 | 0 | 0.333898933 |
| TCGA-BH-A1FE | 2273 | 1 | 0.390757084 |
| TCGA-AO-A0JG | 798 | 0 | 0.099752913 |
| TCGA-C8-A132 | 383 | 0 | 0.089419586 |
| TCGA-OL-A5D6 | 1104 | 1 | 0.285801861 |
| TCGA-A8-A079 | 274 | 0 | 0.248966597 |
| TCGA-AN-A04A | 90 | 0 | 0.167583576 |
| TCGA-AN-A0XT | 10 | 0 | 0.143135304 |
| TCGA-A8-A09V | 457 | 0 | 0.329102149 |
| TCGA-D8-A1Y0 | 472 | 0 | 0.123714512 |
| TCGA-A8-A06N | 0 | 0 | 0.348265537 |
| TCGA-D8-A1XD | 522 | 0 | 0.176413673 |
| TCGA-A8-A081 | 0 | 0 | 0.103377789 |
| TCGA-AO-A124 | 3506 | 0 | 0.072107968 |
| TCGA-AO-A0JM | 2184 | 0 | 0.140690707 |
| TCGA-A2-A4S0 | 706 | 0 | 0.199184175 |
| TCGA-E2-A14U | 1318 | 0 | 0.328725017 |
| TCGA-BH-A1EU | 1286 | 1 | 0.18811563 |
| TCGA-E2-A1IJ | 865 | 0 | 0.075177731 |
| TCGA-D8-A1JK | 0 | 0 | 0.129699776 |
| TCGA-AC-A5XS | 588 | 0 | 0.199956211 |
| TCGA-A2-A0YT | 723 | 1 | 0.295929967 |
| TCGA-BH-A0B4 | 1191 | 0 | 0.096556818 |
| TCGA-BH-A0BL | 2278 | 0 | 0.054902968 |
| TCGA-AR-A24R | 2653 | 0 | 0.180519098 |
| TCGA-S3-AA12 | 467 | 0 | 0.198777996 |
| TCGA-AR-A251 | 2387 | 0 | 0.154410058 |
| TCGA-E9-A22D | 1248 | 0 | 0.090333329 |
| TCGA-A2-A0T5 | 531 | 0 | 0.147165363 |
| TCGA-A8-A09N | 31 | 0 | 0.134613632 |
| TCGA-4H-AAAK | 348 | 0 | 0.171543872 |
| TCGA-A2-A0D3 | 1873 | 0 | 0.089194402 |
| TCGA-A2-A3KC | 635 | 0 | 0.201590654 |
| TCGA-E2-A1II | 1025 | 0 | 0.080504909 |
| TCGA-E2-A15H | 393 | 0 | 0.034422539 |
| TCGA-A7-A0CD | 1165 | 0 | 0.247911386 |
| TCGA-E2-A155 | 640 | 0 | 0.166809773 |
| TCGA-A7-A4SD | 441 | 0 | 0.05680408 |
| TCGA-AC-A2QH | 1005 | 0 | 0 |
| TCGA-A2-A3XX | 1439 | 1 | 0.138041581 |
| TCGA-AR-A1AV | 1864 | 0 | 0.12207086 |
| TCGA-AN-A0XU | 10 | 0 | 0.129922495 |
| TCGA-A1-A0SB | 259 | 0 | 0.295213209 |
| TCGA-LD-A66U | 422 | 0 | 0.061269936 |
| TCGA-E2-A1L8 | 2240 | 0 | 0.020660149 |
| TCGA-B6-A0WS | 2965 | 1 | 0.069915135 |
| TCGA-A7-A26J | 627 | 0 | 0.331551717 |
| TCGA-AC-A3YI | 707 | 0 | 0.078885679 |
| TCGA-A8-A099 | 304 | 0 | 0.292929963 |
| TCGA-A8-A090 | 0 | 0 | 0.229382323 |
| TCGA-BH-A202 | 795 | 0 | 0.249579703 |
| TCGA-BH-A0E0 | 134 | 0 | 0.09639053 |
| TCGA-AN-A0AK | 224 | 0 | 0.251222986 |
| TCGA-A2-A0CV | 3011 | 0 | 0.148301823 |
| TCGA-AR-A0TQ | 2784 | 0 | 0.100631335 |
| TCGA-BH-A0E9 | 2489 | 0 | 0.115332033 |
| TCGA-AC-A7VB | 250 | 0 | 0.201638812 |
| TCGA-AN-A0FW | 11 | 0 | 0.191372157 |
| TCGA-A2-A0D4 | 767 | 0 | 0.251909627 |
| TCGA-E2-A10B | 1141 | 0 | 0.192230554 |
| TCGA-BH-A1ET | 2520 | 1 | 0.165205374 |
| TCGA-E2-A14T | 2311 | 0 | 0.103892945 |
| TCGA-A7-A4SB | 418 | 0 | 0.158104291 |
| TCGA-AR-A2LH | 616 | 1 | 0.096245641 |
| TCGA-LD-A7W6 | 404 | 0 | 0.11988082 |
| TCGA-E9-A22B | 692 | 0 | 0.165902674 |
| TCGA-C8-A135 | 393 | 0 | 0.130375596 |
| TCGA-EW-A1IY | 258 | 0 | 0.088075728 |
| TCGA-C8-A274 | 508 | 0 | 0.263208563 |
| TCGA-AR-A0TR | 160 | 1 | 0.133316363 |
| TCGA-AC-A8OP | 614 | 0 | 0.149237178 |
| TCGA-AR-A24K | 1548 | 0 | 0.192960488 |
| TCGA-AN-A03Y | 10 | 0 | 0.082860118 |
| TCGA-EW-A1P5 | 703 | 0 | 0.319539223 |
| TCGA-AR-A1AI | 3296 | 0 | 0.099597053 |
| TCGA-C8-A273 | 513 | 0 | 0.14702245 |
| TCGA-A8-A07I | 426 | 0 | 0.179019015 |
| TCGA-D8-A1JC | 480 | 0 | 0.124254981 |
| TCGA-EW-A1PH | 607 | 0 | 0.070146631 |
| TCGA-C8-A12V | 385 | 0 | 0.107031537 |
| TCGA-AN-A0FZ | 10 | 0 | 0.210961989 |
| TCGA-E9-A1NF | 1072 | 1 | 0.293782246 |
| TCGA-AN-A0AR | 10 | 0 | 0.162493233 |
| TCGA-E9-A1N8 | 1039 | 0 | 0.122253156 |
| TCGA-BH-A0H5 | 1620 | 0 | 0.061000988 |
| TCGA-A1-A0SD | 437 | 0 | 0.150502453 |
| TCGA-A8-A08X | 1308 | 0 | 0.174822695 |
| TCGA-AR-A24Q | 3088 | 0 | 0.096225974 |
| TCGA-BH-A0BT | 2365 | 0 | 0.08446271 |
| TCGA-AR-A1AJ | 2383 | 0 | 0.167569631 |
| TCGA-A2-A0YL | 1474 | 0 | 0.116409733 |
| TCGA-A8-A07J | 365 | 0 | 0.18681176 |
| TCGA-C8-A12N | 358 | 0 | 0.157239559 |
| TCGA-A8-A08F | 1004 | 0 | 0.210263432 |
| TCGA-B6-A0RQ | 4267 | 1 | 0.257955125 |
| TCGA-AO-A0J6 | 1140 | 0 | 0.080715616 |
| TCGA-AN-A0XL | 163 | 0 | 0.280957569 |
| TCGA-A2-A04W | 3102 | 0 | 0.129629193 |
| TCGA-D8-A73W | 244 | 0 | 0.21783085 |
| TCGA-B6-A0IB | 3941 | 1 | 0.087868415 |
| TCGA-E2-A9RU | 0 | 0 | 0.139097194 |
| TCGA-A2-A0CS | 2348 | 1 | 0.118683054 |
| TCGA-S3-AA15 | 421 | 0 | 0.065894337 |
| TCGA-BH-A28O | 1120 | 0 | 0.036721082 |
| TCGA-BH-A1FM | 1388 | 1 | 0.272966002 |
| TCGA-A8-A09I | 1371 | 0 | 0.209862676 |
| TCGA-E2-A1IN | 675 | 0 | 0.271658491 |
| TCGA-E2-A1BC | 501 | 0 | 0.116036976 |
| TCGA-OL-A5RX | 878 | 0 | 0.077383871 |
| TCGA-E2-A152 | 1994 | 0 | 0.235985725 |
| TCGA-B6-A0WV | 2417 | 1 | 0.169113035 |
| TCGA-E9-A1RA | 976 | 0 | 0.110014731 |
| TCGA-A8-A07S | 243 | 0 | 0.293153213 |
| TCGA-D8-A1XB | 552 | 0 | 0.203979233 |
| TCGA-AR-A1AS | 1150 | 0 | 0.326447205 |
| TCGA-B6-A1KC | 1326 | 0 | 0.346950601 |
| TCGA-BH-A0EI | 1926 | 0 | 0.317949547 |
| TCGA-C8-A8HR | 408 | 0 | 0.248613074 |
| TCGA-D8-A141 | 626 | 0 | 0.08721782 |
| TCGA-AO-A0JE | 2335 | 0 | 0.146833682 |
| TCGA-BH-A1FG | 577 | 1 | 0.175433786 |
| TCGA-AC-A6IV | 568 | 0 | 0.243322167 |
| TCGA-A2-A0T2 | 255 | 1 | 0.167252515 |
| TCGA-A7-A6VV | 313 | 0 | 0.084916686 |
| TCGA-D8-A1JT | 405 | 0 | 0.270535437 |
| TCGA-A1-A0SM | 242 | 0 | 0.272052399 |
| TCGA-A2-A0YK | 588 | 0 | 0.063485639 |
| TCGA-BH-A18P | 921 | 1 | 0.129079348 |
| TCGA-E9-A226 | 1038 | 0 | 0.215736121 |
| TCGA-OL-A5RY | 752 | 0 | 0.133241882 |
| TCGA-D8-A1XO | 1682 | 0 | 0.270566168 |
| TCGA-BH-A42U | 3364 | 0 | 0.079799132 |
| TCGA-EW-A2FS | 1604 | 0 | 0.055643415 |
| TCGA-A1-A0SG | 434 | 0 | 0.185137524 |
| TCGA-A2-A25C | 523 | 0 | 0.174289577 |
| TCGA-BH-A201 | 856 | 0 | 0.079261771 |
| TCGA-E9-A22E | 912 | 0 | 0.134176554 |
| TCGA-OL-A66N | 792 | 0 | 0.162438846 |
| TCGA-BH-A0DO | 1644 | 0 | 0.099087556 |
| TCGA-B6-A0IO | 5042 | 0 | 0.095270412 |
| TCGA-D8-A145 | 410 | 0 | 0.095044634 |
| TCGA-A7-A0CE | 1074 | 0 | 0.105909941 |
| TCGA-BH-A0EA | 991 | 1 | 0.1245467 |
| TCGA-AO-A1KO | 622 | 0 | 0.234294221 |
| TCGA-A7-A4SE | 644 | 0 | 0.091002028 |
| TCGA-E9-A1RI | 1084 | 0 | 0.216573042 |
| TCGA-GM-A3XG | 1330 | 0 | 0.171388636 |
| TCGA-BH-A0BR | 2330 | 0 | 0.203730759 |
| TCGA-A8-A09A | 304 | 0 | 0.102065611 |
| TCGA-AR-A0TU | 709 | 0 | 0.144757663 |
| TCGA-BH-A0C1 | 1411 | 1 | 0.11169763 |
| TCGA-B6-A0RL | 2469 | 1 | 0.246823842 |
| TCGA-AR-A252 | 2226 | 0 | 0.132268478 |
| TCGA-S3-AA11 | 421 | 0 | 0.174745814 |
| TCGA-A8-A082 | 549 | 0 | 0.437155873 |
| TCGA-AR-A0U0 | 1988 | 0 | 0.11115326 |
| TCGA-GM-A2DC | 2535 | 0 | 0.178751878 |
| TCGA-AR-A24L | 2866 | 1 | 0.201438902 |
| TCGA-D8-A1JU | 447 | 0 | 0.311748519 |
| TCGA-E2-A1LH | 3247 | 0 | 0.093370978 |
| TCGA-AN-A0AT | 10 | 0 | 0.096550871 |
| TCGA-AO-A0JB | 1542 | 0 | 0.155712568 |
| TCGA-BH-A1FD | 1009 | 1 | 0.357344421 |
| TCGA-D8-A1XZ | 466 | 0 | 0.196225167 |
| TCGA-BH-A5IZ | 567 | 0 | 0.193178591 |
| TCGA-C8-A131 | 411 | 0 | 0.160292929 |
| TCGA-A7-A2KD | 679 | 0 | 0.173333464 |
| TCGA-B6-A0I6 | 991 | 1 | 0.120319181 |
| TCGA-BH-A42T | 320 | 1 | 0.123680917 |
| TCGA-B6-A0IP | 3926 | 1 | 0.052451024 |
| TCGA-BH-A0DP | 476 | 0 | 0.141643421 |
| TCGA-C8-A1HG | 345 | 0 | 0.186845965 |
| TCGA-D8-A27G | 409 | 0 | 0.143332417 |
| TCGA-E9-A295 | 375 | 0 | 0.157112503 |
| TCGA-AC-A3TM | 762 | 0 | 0.166186992 |
| TCGA-BH-A0BC | 974 | 0 | 0.106072957 |
| TCGA-AN-A03X | 10 | 0 | 0.271890737 |
| TCGA-AO-A12C | 2372 | 0 | 0.143333389 |
| TCGA-AO-A1KP | 2953 | 0 | 0 |
| TCGA-A7-A13E | 614 | 1 | 0.131402588 |
| TCGA-D8-A1X6 | 541 | 0 | 0.482295651 |
| TCGA-C8-A1HM | 375 | 0 | 0.131768718 |
| TCGA-D8-A27M | 410 | 0 | 0.12563109 |
| TCGA-EW-A1IX | 1208 | 0 | 0.141004414 |
| TCGA-B6-A0IE | 1993 | 1 | 0.088526932 |
| TCGA-BH-A0DE | 2372 | 0 | 0.235472491 |
| TCGA-E2-A14Q | 1163 | 0 | 0.101388903 |
| TCGA-BH-A0DZ | 495 | 0 | 0.272367956 |
| TCGA-E2-A15F | 658 | 0 | 0.104205906 |
| TCGA-D8-A27W | 373 | 0 | 0.185308983 |
| TCGA-E2-A158 | 450 | 0 | 0.107077072 |
| TCGA-AN-A049 | 19 | 0 | 0.136583948 |
| TCGA-AR-A0TT | 2636 | 0 | 0.075068203 |
| TCGA-EW-A1P1 | 1210 | 0 | 0.114860207 |
| TCGA-LL-A6FQ | 80 | 0 | 0.247315912 |
| TCGA-PE-A5DC | 1430 | 1 | 0.094461998 |
| TCGA-GM-A3NW | 3361 | 0 | 0.112461217 |
| TCGA-EW-A1P8 | 239 | 1 | 0.255009429 |
| TCGA-EW-A1PF | 439 | 0 | 0.134489391 |
| TCGA-LL-A6FR | 489 | 0 | 0.200820149 |
| TCGA-D8-A1XG | 448 | 0 | 0.211040788 |
| TCGA-EW-A2FV | 788 | 0 | 0.139376974 |
| TCGA-PL-A8LZ | 302 | 0 | 0.104841119 |
| TCGA-BH-A0RX | 170 | 0 | 0.110326711 |
| TCGA-BH-A1FB | 3669 | 1 | 0.105065586 |
| TCGA-WT-AB41 | 1611 | 0 | 0.091675377 |
| TCGA-AO-A0JD | 2190 | 0 | 0.13620189 |
| TCGA-D8-A142 | 425 | 0 | 0.117452155 |
| TCGA-A2-A1G1 | 584 | 0 | 0.187972342 |
| TCGA-LD-A74U | 402 | 0 | 0.152297414 |
| TCGA-E2-A14R | 1174 | 0 | 0.063342857 |
| TCGA-B6-A0RU | 8605 | 0 | 0.165093881 |
| TCGA-A2-A0T1 | 521 | 0 | 0.166588597 |
| TCGA-AR-A255 | 1792 | 0 | 0.173025212 |
| TCGA-D8-A1XM | 538 | 0 | 0.192671203 |
| TCGA-E9-A1NH | 576 | 0 | 0.122403301 |
| TCGA-OL-A66I | 714 | 0 | 0.058862938 |
| TCGA-BH-A0AV | 1820 | 0 | 0.117188061 |
| TCGA-A2-A0YC | 990 | 0 | 0.149292416 |
| TCGA-E9-A1N5 | 1120 | 0 | 0.140401219 |
| TCGA-BH-A0HF | 727 | 0 | 0.133140948 |
| TCGA-D8-A1XW | 1309 | 0 | 0.312621985 |
| TCGA-A1-A0SO | 852 | 0 | 0.12712385 |
| TCGA-Z7-A8R5 | 3287 | 0 | 0.162004736 |
| TCGA-A2-A3XY | 1064 | 0 | 0.067075613 |
| TCGA-BH-A0B7 | 2559 | 0 | 0.069735071 |
| TCGA-GM-A5PV | 412 | 0 | 0.167419042 |
| TCGA-E2-A1B4 | 1004 | 1 | 0.128186405 |
| TCGA-C8-A138 | 380 | 0 | 0.075039656 |
| TCGA-AC-A3QP | 675 | 0 | 0.166901631 |
| TCGA-E9-A1R0 | 860 | 0 | 0.122644112 |
| TCGA-D8-A27P | 49 | 0 | 0.157548266 |
| TCGA-BH-A0DG | 2041 | 0 | 0.193775953 |
| TCGA-B6-A0IG | 4456 | 1 | 0.051882194 |
| TCGA-AN-A0AL | 227 | 0 | 0.086519753 |
| TCGA-A7-A13G | 718 | 0 | 0.35810752 |
| TCGA-AR-A24T | 2312 | 0 | 0.236890752 |
| TCGA-C8-A1HE | 375 | 0 | 0.204497625 |
| TCGA-D8-A27E | 530 | 0 | 0.217815329 |
| TCGA-AQ-A04H | 754 | 0 | 0.182696318 |
| TCGA-B6-A0IM | 3873 | 1 | 0.200770723 |
| TCGA-B6-A0RT | 2721 | 0 | 0.037481386 |
| TCGA-BH-A0BQ | 2255 | 0 | 0.077830892 |
| TCGA-BH-A18G | 149 | 0 | 0.186088675 |
| TCGA-E9-A2JS | 904 | 1 | 0.20607767 |
| TCGA-AN-A0FD | 196 | 0 | 0.121325174 |
| TCGA-A1-A0SE | 1321 | 0 | 0.082063527 |
| TCGA-AC-A23C | 585 | 0 | 0.170649162 |
| TCGA-E2-A10E | 865 | 0 | 0.086909105 |
| TCGA-GM-A2DK | 2645 | 0 | 0.200393193 |
| TCGA-BH-A18M | 2207 | 1 | 0.179130469 |
| TCGA-AN-A0XR | 10 | 0 | 0.148277553 |
| TCGA-A1-A0SP | 584 | 0 | 0.098487397 |
| TCGA-A8-A08H | 0 | 0 | 0.365462454 |
| TCGA-BH-AB28 | 287 | 0 | 0.144416885 |
| TCGA-3C-AALK | 1221 | 0 | 0.150348938 |
| TCGA-BH-A208 | 1759 | 1 | 0.203292411 |
| TCGA-C8-A1HO | 375 | 0 | 0.167142086 |
| TCGA-A8-A085 | 1124 | 0 | 0.239563643 |
| TCGA-AC-A2BM | 3022 | 0 | 0.130038524 |
| TCGA-BH-A0HX | 829 | 0 | 0.118939685 |
| TCGA-B6-A400 | 215 | 0 | 0.056750422 |
| TCGA-A7-A0CG | 1043 | 0 | 0.115169428 |
| TCGA-B6-A409 | 573 | 1 | 0.118938724 |
| TCGA-A2-A0SV | 825 | 1 | 0.290180195 |
| TCGA-OL-A66J | 1996 | 0 | 0.164929198 |
| TCGA-E2-A3DX | 478 | 0 | 0.101697615 |
| TCGA-E9-A22G | 1239 | 0 | 0.080832668 |
| TCGA-A2-A0EX | 752 | 0 | 0.116738371 |
| TCGA-E2-A106 | 2541 | 0 | 0.165370772 |
| TCGA-E2-A572 | 1208 | 0 | 0.094276389 |
| TCGA-OL-A97C | 271 | 0 | 0.131525551 |
| TCGA-AR-A24U | 2363 | 0 | 0.157162104 |
| TCGA-D8-A1JL | 611 | 0 | 0.12627512 |
| TCGA-UU-A93S | 116 | 1 | 0.121477165 |
| TCGA-E2-A15P | 595 | 0 | 0.126034163 |
| TCGA-A7-A13F | 765 | 0 | 0.294627546 |
| TCGA-AR-A5QP | 622 | 0 | 0.131623646 |
| TCGA-OL-A6VO | 858 | 0 | 0.081722516 |
| TCGA-AR-A24S | 2976 | 0 | 0.163744578 |
| TCGA-AC-A62V | 348 | 1 | 0.293536559 |
| TCGA-A8-A09Q | 761 | 0 | 0.219587343 |
| TCGA-A8-A0A7 | 30 | 0 | 0.090218276 |
| TCGA-A8-A08B | 1156 | 0 | 0.08337253 |
| TCGA-B6-A1KN | 4233 | 0 | 0.133032772 |
| TCGA-AR-A2LM | 1585 | 0 | 0.114096722 |
| TCGA-E2-A15E | 630 | 0 | 0.138216093 |
| TCGA-B6-A0I8 | 749 | 1 | 0.301755637 |
| TCGA-AO-A0J2 | 997 | 0 | 0.142375607 |
| TCGA-E9-A1R3 | 78 | 0 | 0.171195724 |
| TCGA-B6-A0RI | 7126 | 0 | 0.254949027 |
| TCGA-A2-A0EW | 1884 | 1 | 0.178481841 |
| TCGA-A2-A0EM | 3094 | 0 | 0.146795971 |
| TCGA-D8-A1X8 | 783 | 0 | 0.188294979 |
| TCGA-A2-A1G6 | 501 | 0 | 0.232132372 |
| TCGA-D8-A1XF | 463 | 0 | 0.193285844 |
| TCGA-EW-A3E8 | 1035 | 0 | 0.059840286 |
| TCGA-BH-A0HW | 1561 | 0 | 0.25877468 |
| TCGA-A2-A0T6 | 575 | 0 | 0.133411921 |
| TCGA-EW-A1PG | 1051 | 0 | 0.110730619 |
| TCGA-A2-A3Y0 | 1167 | 0 | 0.063173697 |
| TCGA-E2-A1B0 | 1631 | 0 | 0.113532652 |
| TCGA-A8-A09K | 912 | 0 | 0.124878723 |
| TCGA-E9-A1R4 | 186 | 0 | 0.210967439 |
| TCGA-E2-A156 | 726 | 0 | 0.298303982 |
| TCGA-AO-A03M | 1866 | 0 | 0.114164855 |
| TCGA-E2-A15O | 289 | 0 | 0.074420429 |
| TCGA-A7-A4SF | 545 | 0 | 0.102593972 |
| TCGA-C8-A27A | 747 | 0 | 0.141340081 |
| TCGA-AC-A2B8 | 677 | 0 | 0.157967537 |
| TCGA-E9-A228 | 1285 | 0 | 0.201439834 |
| TCGA-AR-A1AQ | 3021 | 0 | 0.125081026 |
| TCGA-AR-A0TV | 2288 | 0 | 0.109255362 |
| TCGA-A2-A0CQ | 2695 | 0 | 0.385221022 |
| TCGA-AN-A0FY | 10 | 0 | 0.179587591 |
| TCGA-D8-A1JN | 620 | 0 | 0.167118139 |
| TCGA-E9-A245 | 26 | 0 | 0.277971837 |
| TCGA-BH-A1FH | 1034 | 1 | 0.201891503 |
| TCGA-EW-A1IZ | 554 | 0 | 0.062837463 |
| TCGA-D8-A1J9 | 532 | 0 | 0.275516464 |
| TCGA-A2-A0CK | 4159 | 0 | 0.095065422 |
| TCGA-BH-A0DX | 2156 | 0 | 0.148053172 |
| TCGA-A8-A097 | 365 | 0 | 0.196158427 |
| TCGA-5L-AAT0 | 1477 | 0 | 0.114349331 |
| TCGA-BH-A1F5 | 2712 | 1 | 0.266084129 |
| TCGA-B6-A0RS | 3063 | 1 | 0.16856574 |
| TCGA-BH-A0E7 | 1363 | 0 | 0.329464044 |
| TCGA-E9-A2JT | 288 | 0 | 0.021273032 |
| TCGA-AR-A1AK | 3159 | 0 | 0.186212568 |
| TCGA-BH-A42V | 635 | 0 | 0.094892735 |
| TCGA-A8-A06Q | 31 | 0 | 0.365322062 |
| TCGA-BH-A18F | 1001 | 0 | 0.100595844 |
| TCGA-OL-A66L | 1301 | 0 | 0.168913055 |
| TCGA-A2-A25A | 3276 | 0 | 0.13988197 |
| TCGA-A8-A06R | 547 | 0 | 0.161721024 |
| TCGA-D8-A1JJ | 611 | 0 | 0.188297436 |
| TCGA-E2-A1IK | 1800 | 0 | 0.115455716 |
| TCGA-AR-A2LO | 935 | 0 | 0.104780932 |
| TCGA-AR-A24V | 2596 | 0 | 0.287902804 |
| TCGA-PL-A8LX | 5 | 0 | 0.440502144 |
| TCGA-GI-A2C8 | 225 | 0 | 0.268076669 |
| TCGA-A2-A0SU | 1662 | 0 | 0.080623319 |
| TCGA-C8-A12Q | 385 | 1 | 0.189438566 |
| TCGA-B6-A0RN | 8008 | 0 | 0.163463021 |
| TCGA-A2-A0CR | 3283 | 0 | 0.059992532 |
| TCGA-BH-A0HO | 76 | 0 | 0.28324756 |
| TCGA-E2-A1LB | 2306 | 0 | 0.252211549 |
| TCGA-A1-A0SF | 1463 | 0 | 0.069022565 |
| TCGA-E9-A1ND | 838 | 0 | 0.157834617 |
| TCGA-AO-A03O | 2483 | 1 | 0.153878511 |
| TCGA-C8-A12K | 0 | 0 | 0.066359892 |
| TCGA-EW-A1IW | 371 | 0 | 0.129903264 |
| TCGA-D8-A1JS | 371 | 0 | 0.175288706 |
| TCGA-A8-A07R | 273 | 0 | 0.153609732 |
| TCGA-AO-A0J5 | 792 | 1 | 0.186369566 |
| TCGA-B6-A1KI | 2236 | 0 | 0.156967695 |
| TCGA-A2-A0EO | 2442 | 0 | 0.111077609 |
| TCGA-AR-A1AR | 524 | 1 | 0.185264007 |
| TCGA-E2-A108 | 837 | 0 | 0.092379415 |
| TCGA-E2-A10F | 878 | 0 | 0.110958944 |
| TCGA-B6-A0X5 | 2097 | 1 | 0.321369467 |
| TCGA-EW-A1P6 | 562 | 0 | 0.200370422 |
| TCGA-AR-A2LE | 4239 | 0 | 0.172834192 |
| TCGA-BH-A1F2 | 959 | 1 | 0.187245398 |
| TCGA-BH-A0W5 | 1288 | 0 | 0.047558398 |
| TCGA-AR-A0TS | 2275 | 0 | 0.101293379 |
| TCGA-AN-A0AJ | 303 | 0 | 0.132234621 |
| TCGA-E2-A15G | 554 | 0 | 0.239692172 |
| TCGA-A2-A0ST | 3017 | 0 | 0.029763532 |
| TCGA-D8-A27F | 488 | 0 | 0.107318869 |
| TCGA-C8-A1HF | 332 | 0 | 0.202179465 |
| TCGA-AR-A0U4 | 2615 | 0 | 0.086028665 |
| TCGA-GM-A2DA | 6593 | 1 | 0.039773447 |
| TCGA-AR-A5QM | 1665 | 0 | 0.116106373 |
| TCGA-BH-A0H6 | 747 | 0 | 0.403600531 |
| TCGA-E2-A15M | 336 | 1 | 0.170069475 |
| TCGA-AO-A03P | 2911 | 1 | 0.21437178 |
| TCGA-B6-A0RV | 5156 | 0 | 0.128468259 |
| TCGA-A8-A07C | 1034 | 0 | 0.220875868 |
| TCGA-LL-A440 | 759 | 0 | 0.154424114 |
| TCGA-B6-A0I1 | 2361 | 1 | 0.068323606 |
| TCGA-BH-A0HP | 414 | 0 | 0.219214025 |
| TCGA-D8-A1JI | 577 | 0 | 0.359621153 |
| TCGA-E9-A3X8 | 478 | 0 | 0.079984556 |
| TCGA-EW-A1PE | 320 | 0 | 0.156340423 |
| TCGA-AR-A24N | 2388 | 0 | 0.171595238 |
| TCGA-A2-A1FV | 714 | 0 | 0.2395099 |
| TCGA-AR-A0U3 | 3468 | 0 | 0.085274254 |
| TCGA-A2-A0EP | 3273 | 0 | 0.080165655 |
| TCGA-AO-A12A | 3112 | 0 | 0.069540102 |
| TCGA-A7-A3J1 | 343 | 0 | 0.127719695 |
| TCGA-BH-A0AU | 1914 | 0 | 0.097311115 |
| TCGA-BH-A0BA | 1132 | 0 | 0.051970195 |
| TCGA-AN-A0AS | 10 | 0 | 0.138625266 |
| TCGA-A2-A04Y | 1099 | 0 | 0.254591252 |
| TCGA-A8-A09R | 273 | 0 | 0.158546417 |

Abbreviations: Id=Patient Number

Futime=date of follow-up

Fustate=vital status ,0=death,1=alive

Macrophages M2=Macrophage M2 infiltration values
